# Enhancing biofuels production by engineering the actin cytoskeleton in *Saccharomyces cerevisiae*

**DOI:** 10.1038/s41467-022-29560-6

**Published:** 2022-04-07

**Authors:** Hui Liu, Pei Zhou, Mengya Qi, Liang Guo, Cong Gao, Guipeng Hu, Wei Song, Jing Wu, Xiulai Chen, Jian Chen, Wei Chen, Liming Liu

**Affiliations:** 1 State Key Laboratory of Food Science and Technology, Jiangnan University, Wuxi, 214122 China; 2 Key Laboratory of Industrial Biotechnology, Ministry of Education, Jiangnan University, Wuxi, 214122 China; 3 School of Pharmaceutical Science, Jiangnan University, 1800 Lihu Road, Wuxi, 214122 China

**Keywords:** Metabolic engineering, Metabolic engineering, Applied microbiology

## Abstract

*Saccharomyces cerevisiae* is widely employed as a cell factory for the production of biofuels. However, product toxicity has hindered improvements in biofuel production. Here, we engineer the actin cytoskeleton in *S. cerevisiae* to increase both the cell growth and production of n-butanol and medium-chain fatty acids. Actin cable tortuosity is regulated using an n-butanol responsive promoter-based autonomous bidirectional signal conditioner in *S. cerevisiae*. The budding index is increased by 14.0%, resulting in the highest n-butanol titer of 1674.3 mg L^−1^. Moreover, actin patch density is fine-tuned using a medium-chain fatty acid responsive promoter-based autonomous bidirectional signal conditioner. The intracellular pH is stabilized at 6.4, yielding the highest medium-chain fatty acids titer of 692.3 mg L^−1^ in yeast extract peptone dextrose medium. Engineering the actin cytoskeleton in *S. cerevisiae* can efficiently alleviate biofuels toxicity and enhance biofuels production.

## Abstract (web summary)

Product toxicity is one of the factors that hinder biofuel production. Here, the authors engineer the actin cytoskeleton to increase cell growth and production of n-butanol and medium-chain fatty acids in *Saccharomyces cerevisiae*.

## Introduction

*Saccharomyces cerevisiae* is a workhorse for the production of large amounts of biofuels, such as ethanol, n-butanol, isobutanol, and fatty acids from various feedstocks^1^. Owing to its high tolerance to harsh fermentation conditions and relative ease of genetic manipulation^2^, *S. cerevisiae* has been engineered to produce biofuels, such as short-chain alcohols and medium-chain fatty acids (MCFAs)^3,4^. For example, *S. cerevisiae* can produce 1.05 g L^−1^ n-butanol and 1.39 g L^−1^ MCFAs after the engineering of its native 2-keto-acid pathway^5^ and fatty acid synthases^6^, respectively. However, there are limits on further increases in titer, yield, and productivity of biofuels because the elevated hydrophobicity and lipophilicity of biofuels make them toxic to yeast^3^.

Various strategies, such as evolution engineering, transcriptional factor engineering, and efflux pump engineering, have been attempted to further increase the titer, yield, and productivity of biofuels in *S. cerevisiae*. In evolutionary engineering, generating and taking advantage of plasticity of the genome is key to obtaining robust microbial cell factories, which can then be refined by adaptive laboratory evolution^7^ and artificial synthetic evolution^8^. Adaptive laboratory evolution has been widely applied to increase the stress tolerance of yeast to biofuels, such as MCFAs^6^, n-hexanol^9^, isobutanol^10^, and isoprenol^11^. Moreover, the rapid development of DNA synthesis methodologies^8^, multiplex automated genome evolution^12^, trackable multiplex recombineering^13^, recombinase-mediated evolution^14^, and CRISPR-mediated genomic editing technology^15^ have allowed direct evolution of microbial genomes as a means of augmenting tolerance to biofuels. For instance, after applying a random recombination strategy to assemble multiple stress defense circuits, including heat shock proteins, ubiquitins, antioxidant proteins, and stress-related transcriptional factors, ethanol toxicity to *S. cerevisiae* was alleviated, and ethanol production was increased by 6.9% on an industrial scale^16^. In transcriptional factor engineering, the goal is to regulate a group of genes. This can be achieved by engineering specific transcription factors^17^ and/or global transcription processes^18^. Global transcriptional regulators, such as sigma factors^18^, mediator complex^19^, and histone-like nucleoid structuring factor^20^, have been widely used to enhance biofuel tolerance. For example, engineering sigma factors (RpoD) by random mutagenesis successfully augmented ethanol tolerance in *Zymomonas mobilis* and increased ethanol production by 1.8-fold compared to the control^21^. In efflux pump engineering, the purpose is to relieve cellular toxicity by promoting the export of noxious compounds from the cell. Five families of efflux pumps, including the ATP-binding cassette (ABC) family^22^, the multidrug and toxin extrusion family^23^, the major facilitator superfamily^24^, the small multidrug resistance family^25^, and resistance nodulation division efflux pump family^25^, have been engineered to export the toxic biofuels to alleviate the impact on growth. For example, to reduce the cellular toxicity of alkane, the heterogeneous membrane ABC transporters ABC2 and ABC3 were introduced into *S. cerevisiae*, leading to enhanced alkane tolerance and a 30-fold increase in the export of undecane^26^. Together, these three strategies are efficient in improving the stress tolerance of cellular factories to biofuels; however, they are laborious and limited by the complexity of the stress tolerance mechanism. To further increase stress tolerance, the ways in which biofuels cause damage to the cell need to be elucidated. Knowing these damage patterns will facilitate development of a rational and targeted strategy.

In this work, a rational strategy to improve biofuel tolerance is implemented by targeting the pattern of damage induced by n-butanol and MCFAs. Accordingly, n-butanol stress tolerance and production are enhanced by shortening actin cable tortuosity using an n-butanol responsive promoter-based autonomous bidirectional signal conditioner. MCFA stress tolerance and production are also elevated by increasing the actin patch density using an MCFA responsive promoter-based autonomous bidirectional signal conditioner. We demonstrate that this strategy has widespread potential to increase stress tolerance and improve the production of biofuels in yeast.

## Results

### Biofuels cause defects in cell growth of *S. cerevisiae*

**Fig. 1:**
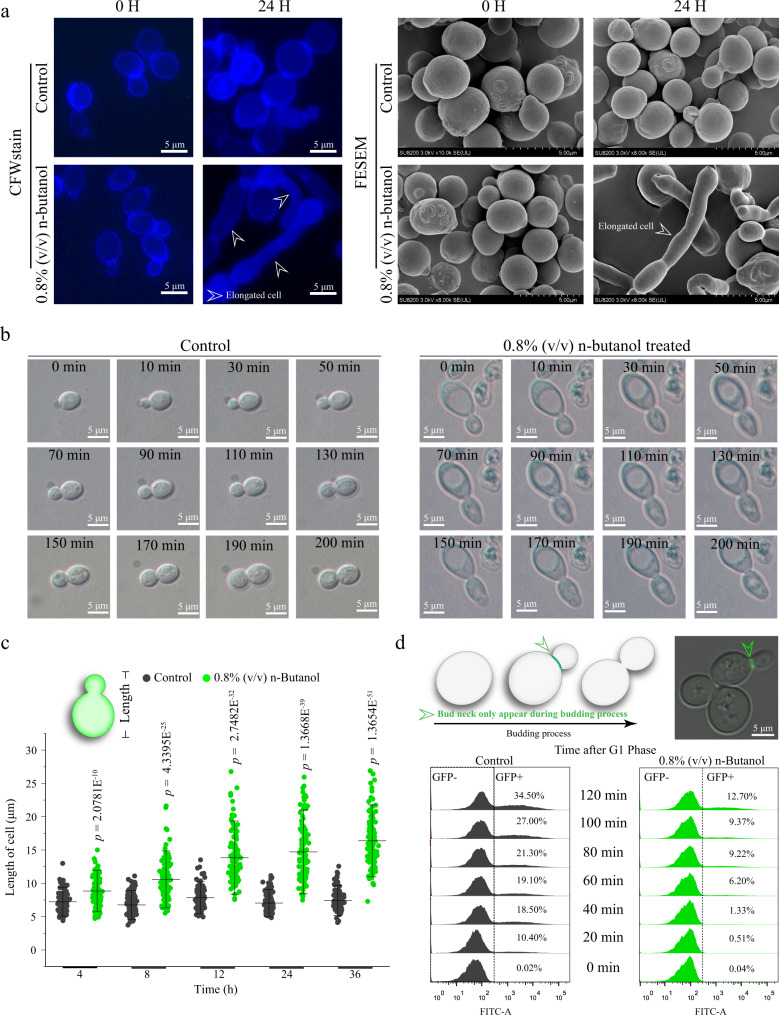
n-Butanol decrease budding index of *S. cerevisiae*. **a** The cell morphology change was detected by microscope and the cells were stained by calcofluor white (CFW). Additionally, field emission scanning electron microscopy (FESEM) was applied to confirm the morphology change under 0.8% (v/v) n-butanol treatment. The white arrows point to the elongated cells. **b** The time-lapse microscopy of a yeast cell that undergoing the budding process in the control and n-butanol treated group. The yeast cells were acquired at 20 min intervals. Time is indicated in minutes. **c** Cell length was detected by the microscope, and 120 cells were analyzed both in the control and n-butanol treated group. *P* values are from a Student’s two-sided *t*-test of the difference from the untreated control group. Values are shown as mean ± S.D. from 120 (*n* = 120) cells over three independent biological replicates. **d** The budding indexes of the control and n-butanol treated group was measured by FACS. The bud neck protein Cdc10 was used as an indicator that visualized the budding process of *S. cerevisiae*. Here, the fusion protein Cdc10-GFP was introduced into *S. cerevisiae*, and the GFP signal was just appearing at cells that were under budding process. The high GFP ratio indicates a higher budding index. The green arrows point to the bud necks. Values and error bars represent the mean values and standard deviations of biological repeats. Abbreviations: FACS Fluorescence-activated cell sorting; GFP green fluorescent protein; CFW calcofluor white; FESEM field emission scanning electron microscopy. **a**, **b**, **d** Three experiments (*n* = 3) were repeated independently with similar results. Source data are provided as a Source Data file.

Initially, under conditions of 0.8% (v/v) n-butanol, cell growth at the population level as measured by cell density (OD_600_) was decreased by 53.9% (Supplementary Fig. 1a). At the single-cell level, the fraction of viable cells (unstained by propidium iodide) was decreased by 44.4% (Supplementary Fig. 1b). Furthermore, it was found that: (1) the yeast cell became progressively longer and adopted a pseudohyphal morphology (Fig. 1a, c, and Supplementary Fig. 2); (2) the cell cycle was delayed at G1 phase (Supplementary Fig. 3). As budding formation is coordinated with cell cycle^27^, suggesting that the G1 phase delay caused by n-butanol may lead to budding process defects. Based on these, the budding capacity analysis showed that the budding pattern on the single-cell level was damaged (Fig. 1b) and the relative budding index was dropped by 19.8% after treating with 0.8% (v/v) n-butanol (Fig. 1d). The budding process determines the cell growth of *S. cerevisiae*^28,29^, indicating that budding index is one of the critical reasons for cell growth in the presence of n-butanol.

**Fig. 2:**
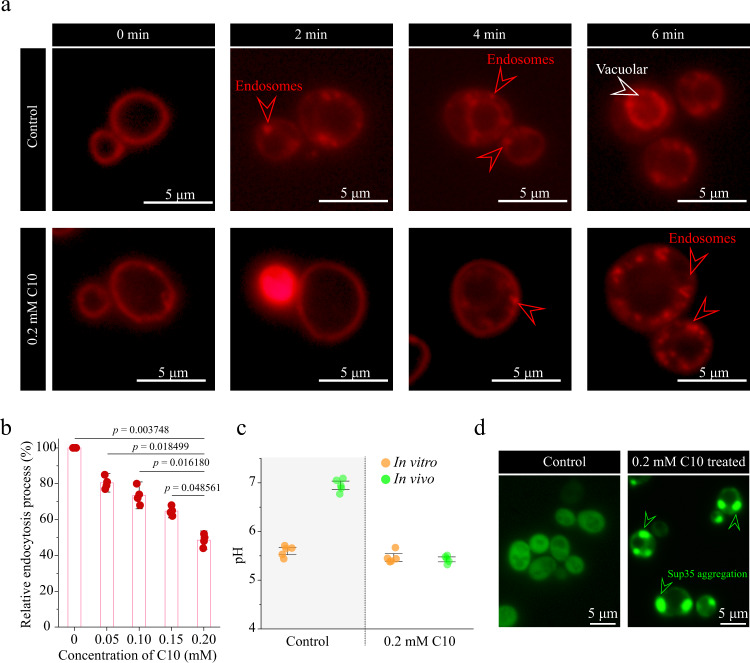
Decanoic acid damage stabilization of pH_i_ of *S. cerevisiae*. **a** Internalization of FM4-64 under 0 mM and 0.2 mM decanoic acid treatment. Cells were incubated with FM4-64 on ice, then the cells of different conditions were released and incubated at 30 ^o^C. The fluorescence images were shown for timepoints of 0, 2, 4, 6 min at 30 ^o^C. The red arrows point toward the endosome and the white one toward the vacuole. The red arrows point to the endosome and the white arrows point to the vacuolar. **b** The internalization of fluorescent unit Cy5 conjugated α-factor in 0 mM and 0.2 mM decanoic acid conditions. The cells were incubated with α-factor at 30 ^o^C and then the ratio endocytosis process was dependent on the internalization of Cy5 conjugated α-factor. The data were analyzed by FACS. Significance (*p*-value) was evaluated by two-sided *t*-test. **c** The comparison of in vivo and in vitro pH values in 0 mM and 0.2 mM decanoic acid treatment. **d** The fluorescence microscope images of *S. cerevisiae* expression Sup35-GFP fusion protein (a protein that just aggregate under low pH_i_) under 0 mM and 0.2 mM decanoic acid treatment. Green arrows point toward Sup35 aggregations. Abbreviation: C10 decanoic acid. All the experiments were performed in three biological repeats. Values and error bars represent the mean values and standard deviations of biological repeats. Source data are provided as a Source Data file.

Next, under growth conditions of 0.2 mM decanoic acid, the cell density (OD_600_) was decreased by 48.4% (Supplementary Fig. 4a), and the fraction of viable cells was decreased by 60.5% (Supplementary Fig. 4b). Further investigation on the decreased cell growth revealed that the presence of 0.2 mM decanoic acid delayed the formation of the endosome and vacuole, as revealed by an analysis with the lipophilic dye FM4-64 (Fig. 2a). Endocytosis was decreased by 47.3%, as revealed by an analysis using the fluorescent unit Cy5-conjugated α-factor (Fig. 2b). Intracellular pH (pH_i_) dropped from 7.0 to 5.4 (Fig. 2c, d and Supplementary Fig. 5). Because maintenance of pH_i_ within physiological limits is vital for survival of *S. cerevisiae* cells, the results suggest that the presence of decanoic acid decreases cell growth via its effects on pH_i_.

### Engineering actin cytoskeleton to increase cell growth

The actin cytoskeleton, especially actin patches and actin cables, plays a fundamental role in cell division and endocytosis^30^. Thus, engineering the actin cytoskeleton has potential as a method to enhance the cell growth of *S. cerevisiae* in the presence of toxic biofuels. Based on our knowledge of the mechanism by which the actin cytoskeleton is formed, 12 genes, encoding components of the polarisome and formin, were selected to tune actin cables^30^, and 8 genes, encoding adapter proteins of patch components, were the potential targets to regulate actin patches^31^.

**Fig. 3:**
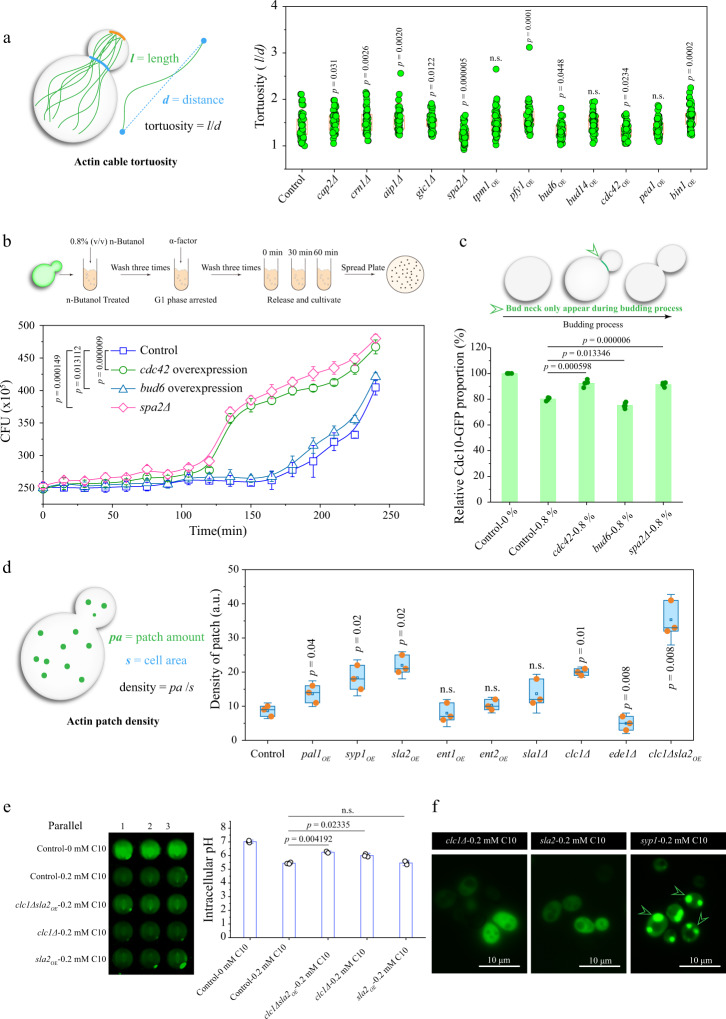
Screening the actin cable genes for increasing budding index of *S. cerevisiae*. **a** The tortuosity is defined as the ratio of the cable length (*l*) to the distance (*d*) between its two endpoints. A total of 12 candidates were observed by fluorescence microscope, and then the tortuosity was analyzed by Image J. Statistical significance were analyzed between control group and different actin cable engineered strains. **b** The stabilization effect of *cdc42*, *spa2*, and *bud6* on the budding index of *S. cerevisiae* under 0.8% (v/v) n-butanol treatment. The budding index was determined by colony-forming unit (CFU). **c** The Cdc10-GFP was applied as a budding indicator to detect the budding process of engineered strains under n-butanol treatment. The GFP signal was just appearing at cells that were under budding process. The high GFP ratio indicates a higher budding index. The green arrows point to the bud necks. Statistical significance were analyzed between control group under 0.8% (v/v) n-butanol treatment and corresponding engineered strains with *spa2Δ*, *cdc42*, and *bud6* overexpression. **d** The fluorescence microscope images of actin patch. The density of the patch determined the endocytosis process of *S. cerevisiae*. The effect of improving the density of the patch was analyzed by fluorescence microscope and the GFP dot indicated the actin patch. The patch was characterized by fusing the GFP with the genomic Abp1. The box plots showing the minima, maxima, center, bounds of box and whiskers and percentile. Statistical significance were analyzed between control group and different actin patch engineered strains. **e** The effect of relieving the decline of intracellular pH. Statistical significance were analyzed between control group under 0.2 mM decanoic acid treatment and corresponding engineered strains with *clc1Δ*, *clc1Δsla2*_*OE*_, and *sla2* overexpression. **f** The fluorescence microscope images of *S. cerevisiae* expression Sup35-GFP fusion protein under 0.2 mM decanoic acid treatment in strain *clc1Δ*, *sla2* overexpression strain, and *syp1* overexpression strain. Green arrows point toward Sup35 aggregations. All experiments were performed in three biological repeats. Values and error bars represent the mean values and standard deviations of biological repeats. **a**, **c**–**e**, Significance (*p*-value) was evaluated by two-sided t-test. Source data are provided as a Source Data file.

Actin cable tortuosity was used as a parameter to screen for budding process regulators because of its role in stabilizing the cell division process^32^. As shown in Fig. 3a, compared to the control strain, *S. cerevisiae* with the *cap2*, *crn1*, *aip1*, *gic1*, or *spa2* gene deleted exhibited an increase in actin cable tortuosity of 7.1%, 10.7%, 11.1%, 6.7%, and −12.5%, respectively. Overexpression of *tpm1*, *pfy1*, *bud6*, *bud14*, *cdc42*, *pea2*, and *bin1* resulted in increases in actin cable tortuosity of 7.7%, 14.8%, −5.7%, 6.1%, −7.0%, −1.3%, and 14.0%, respectively (Fig. 3a and Supplementary Fig. 6). Because reduced tortuosity resulted in reduced cell size and stabilized the budding process^33^, *spa2*, *cdc42*, and *bud6* were selected to investigate the effect on the budding index (Supplementary Figs. 7–8), which was evaluated using two different approaches. First, the yeast cells were released from G1 phase arrest, and the cells were then plated on yeast nitrogen base (YNB) medium containing 0.8% (v/v) n-butanol. The number of colony-forming units (CFUs) was used to indicate the relative budding index. Compared with the control strain, the *spa2* deletion strain, *cdc42* overexpression strain, and *bud6* overexpression strain exhibited an increase in CFUs by 18.4%, 15.4%, and 4.1%, respectively (Fig. 3b). Second, Cdc10-GFP (a fusion between a component of the septin ring and green fluorescent protein) was used as a marker to visualize the budding index of yeast cells. As shown in Fig. 3c, the relative budding indices of Cdc10-GFP in the *SPA2* deletion strain and the *cdc42* overexpression strain were 14.0% and 15.3% higher than that of the control strain in YNB medium with 0.8% (v/v) n-butanol, respectively. The *bud6* overexpression strain showed a 6.1% decrease in budding index under the same conditions. Therefore, *spa2* and *cdc42* were selected as targets to improve the budding index. Furthermore, simultaneous deletion of *spa2* and overexpression of *cdc42* in strains W303-1A, W303-1B, BY4741, BY4742, Cen.PK2-1C, and thTAM in YNB medium with 0.8% (v/v) n-butanol was found to increase the OD_600_ by 88.3%, 79.6%, 53.9%, 104.0%, 106.7%, and 108.6%, respectively (Supplementary Fig. 9a), and increase the budding index by 18.9%, 21.3%, 19.0%, 16.5%, 14.9%, and 19.6%, respectively (Supplementary Fig. 9b).

The density of actin patches impacts endocytosis, which is important for maintaining the function of the vacuole^30^. Therefore, actin patch density was used to screen for pH_i_ stabilizers. Eight potential genes were divided into two groups: the first group contained five overexpressed genes (*pal1*, *syp1*, *sla2*, *ent1*, and *ent2*), and the second group included three deleted genes (*sla1*, *clc1*, and *ede1*). As shown in Fig. 3d, overexpression of *pal1*, *syp1*, *sla2*, and *ent2* led to increases in actin patch density of 57.1%, 110.7%, 152.9%, and 18.8%, respectively, whereas overexpression of *ent1* led to a decrease of 8.1%. On the contrary, the deletion of *sla1* and *clc1* resulted in increases in actin patch density of 57.1% and 129.9%, respectively, whereas deletion of *ede1* resulted in a decrease of 4.3% (Fig. 3d, Supplementary Fig. 10). Based on these data, *clc1*, *sla2*, and *syp1* were selected to investigate their effects on pH_i_. The pH_i_ values of the *clc1δ*, *sla2* overexpression, and *syp1* overexpression strains in the YNB medium containing 0.2 mM decanoic acid were 6.2, 6.0, and 5.5, respectively (Fig. 3e). This result was confirmed by Sup35-GFP aggregation (a translation termination factor and GFP fusion that forms aggregates under conditions of low intracellular pH^34^). The aggregation was found only in the *syp1*-overexpressing strain, in which pH_i_ was decreased, but not the *clc1Δ* strain or the *sla2*-overexpressing strain (Fig. 3f). Therefore, *clc1* and *sla2* are identified as targets for stabilization of pH_i_. When *sla2* was overexpressed along with *clc1Δ* in strains W303-1A, W303-1B, BY4741, BY4742, Cen.PK2-1C, and thTAM grown in YNB medium with 0.2 mM decanoic acid, the OD_600_ increased by 60.3%, 54.7%, 57.8%, 41.3%, 75.8%, and 59.1%, respectively, relative to control strains (Supplementary Fig. 11a), and the pH_i_ was stabilized at 6.3, 6.1, 6.2, 6.2, 6.1, and 6.2, respectively (Supplementary Fig. 11b). However, in the YNB medium without n-butanol or decanoic acid, deleting and overexpressing genes related to actin cables (*spa2Δcdc42*_OE_) and patches (*clc1Δsla2*_OE_) resulted in 13.1% and 12.7% decreases in the OD_600_ values compared with the control strain (Supplementary Fig. 12). These data indicate that the static engineering strategies are not an optimal tool to tune the expression of the actin cytoskeleton.

### Construction of an autonomous bidirectional signal conditioner for manipulating the actin cytoskeleton

**Fig. 4:**
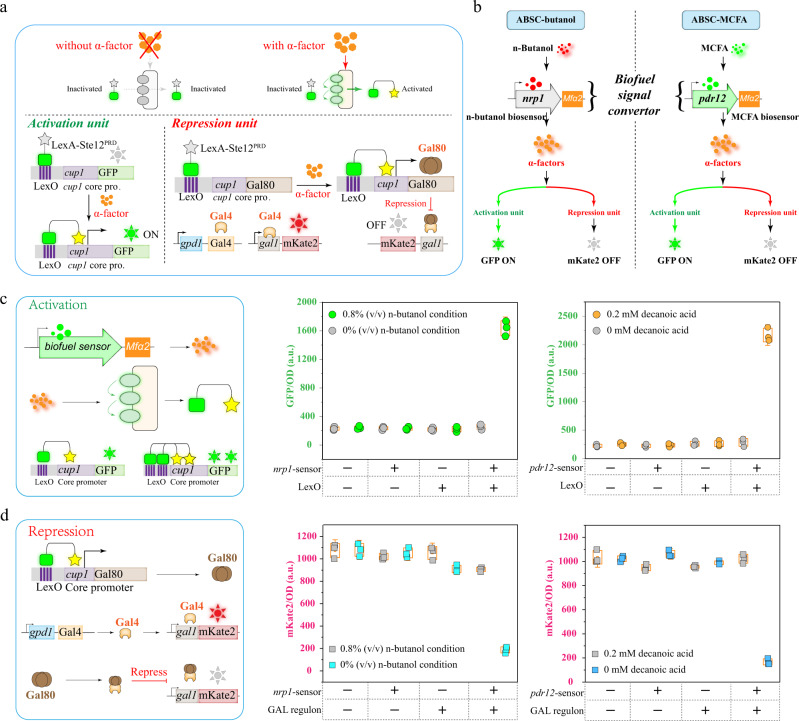
Constructing and characterizing ABSC. **a**, **b** The schematic diagram of the autonomous bidirectional signal conditioner (ABSC). **c** The activation unit of ABSC in biofuels conditions was characterized by GFP. **d** The repression unit of ABSC in biofuels conditions was characterized by mKate2. **c**, **d** Values and error bars represent the mean values and standard deviations of three biological repeats, respectively. Source data are provided as a Source Data file.

To achieve autonomous, temporal, and dual control of the actin cytoskeleton when biofuels accumulate, an autonomous bidirectional signal conditioner (ABSC) containing an activation unit, a repression unit, and a biofuel signal converter was constructed (Fig. 4a, b and Supplementary Fig. 13).

The activation unit consisted of a synthetic transcriptional activator (LexA-Ste12^PRD^), a hybrid LexO-*cup1* promoter comprising a LexA operator (LexO), a *cup1* core promoter sequence, and a GFP reporter gene. LexA-Ste12^PRD^ can be recruited to LexO-*cup1* to allow for transcriptional activation of the GFP reporter. In the absence of α-factor, the yeast mating pheromone response pathway is inactivated, which prevents the activation of LexA-Ste12^PRD^ and the expression of GFP. Conversely, in the presence of the α-factor, LexA-Ste12^PRD^ is activated, which induces the *cup1* promoter and finally activates GFP expression (Fig. 4a). The repression unit was constructed based on the activation unit, the yeast galactose (GAL) regulon composed of transcriptional regulators (Gal80 and Gal4), and the *gal1* promoter was introduced as a “NOT” gate that can turn off gene expression when required. Here, mKate2 is the reporter gene, and Gal80 is expressed under the control of the LexO-*cup1* promoter, which is activated by LexA-Ste12^PRD^. In the absence of α-factor, Gal80 is repressed, so Gal4 can activate mKate2 expression from the *gal1* promoter. Conversely, in the presence of α-factor, Gal80 is expressed, binds to Gal4, and prevents mKate2 expression from the *gal1* promoter (Fig. 4a).

Furthermore, to regulate the activation and repression units simultaneously and autonomously, a biofuel signal converter was constructed. At first, available literature about growth in the presence of n-butanol and MCFAs was mined for transcriptome data^35,36^. From this analysis, potential biofuels responsive promoters were screened. In n-butanol growth conditions, the 15 most differentially expressed genes identified in the transcriptome data were selected, and their promoters were identified as potential n-butanol responsive promoters (Supplementary Fig. 14a). Similarly, 15 potential MCFA responsive promoters were identified from relevant transcriptome data (Supplementary Fig. 14a). Thereafter, to obtain sensors that were tunable, stable, and had a low background of activity, the potential responsive promoters were characterized in a series of different levels of n-butanol or decanoic acid, as appropriate. As a result, *nrp1* and *pdr12* were selected as the n-butanol- and MCFA responsive promoters (Supplementary Fig. 14b), respectively, which showed good specificity for the corresponding biofuel (Supplementary Fig. 14c). Subsequently, the n-butanol-responsive promoter *nrp1* was used to regulate the expression of *mfα2*, the gene encoding α-factor (Fig. 4b and Supplementary Fig. 15). In the presence of n-butanol, *mfa2* is expressed and α-factor is generated, inducing the regulation of the activation and repression units, as described above. Similarly, the MCFA responsive promoter *pdr12* was then used to induce the generation of α-factor and autonomously drive the activation and repression units (Fig. 4b and Supplementary Fig. 15). Based on these results, the corresponding biofuel signal converter, activation unit, and repression unit could thus be assembled to create ABSC-butanol and ABSC-MCFA (Fig. 4b), respectively.

The performance of ABSC-butanol and ABSC-MCFA was evaluated in a series of experiments. With ABSC-butanol, the addition of 0.8% (v/v) n-butanol triggered a 6.2-fold increase in GFP fluorescence intensity driven by the activation unit and a 4.8-fold decrease in mKate2 fluorescence intensity driven by the repression unit compared to the control (Fig. 4c, d). The GFP and mKate2 fluorescence intensities did not change in strains devoid of the *nrp1*-LexO or *nrp1*-GAL regulons, respectively. In ABSC-MCFA, the addition of 0.2 mM decanoic acid led to a 7.6-fold increase in GFP fluorescence intensity driven by the activation unit and a 6.1-fold decrease in mKate2 fluorescence intensity driven by the repression unit compared to the control (Fig. 4c, d). The GFP and mKate2 fluorescence intensities did not change in strains devoid of the *pdr12*-LexO or *pdr12*-GAL regulons, respectively. However, this narrow dynamic range was deemed insufficient to regulate the expression of the actin cytoskeleton. Therefore, to optimize the activation unit, the number of lexO-binding sites (1× to 8× LexO) and the core promoters of LexO-*cup1* were changed. As a result, GFP fluorescence intensity increased 12.7-fold and 16.3-fold following treatment with 0.8% (v/v) n-butanol and 0.2 mM decanoic acid, respectively (Supplementary Fig. 16a). In addition, optimization of the number of LexO-binding sites and the constitutive promoter of Gal4 allowed the mKate2 fluorescence intensity to decrease by 12.0-fold and 16.8-fold following treatment with 0.8% (v/v) n-butanol and 0.2 mM decanoic acid, respectively (Supplementary Fig. 16b). Moreover, functioning of the ABSC system was confirmed at the 5-L bioreactor level (Supplementary Fig. 17). A computational model was constructed to better understand the behavior of ABSC-butanol and ABSC-MCFA, respectively (Supplementary Fig. 18).

To confirm that ABSC could efficiently regulate the expression of endogenous genes, ABSC-butanol and ABSC-MCFA were introduced into strain BY4741 to generate strains LH001-B and LH001-M, respectively, and the fluorescent proteins were replaced by the corresponding genes encoding components of the actin cytoskeleton. Following 0.8% (v/v) n-butanol treatment, compared to the control strain, the budding index and cell density (OD_600_) in strain LH001-B were increased by 1.2- and 1.7-fold, respectively (Supplementary Fig. 19a). Additionally, following 0.2 mM decanoic acid treatment, the pH_i_ of strain LH001-M was maintained at 6.1 and the cell density (OD_600_) was 56.2% higher than that of the control strain (Supplementary Fig. 19b). Furthermore, the universality of ABSC to regulate the expression of the actin cytoskeleton was confirmed in *S. cerevisiae* strains, including W303-1A and Cen.PK2-1C (Supplementary Fig. 20). Overall, these results suggest that ABSC has broad applicability for tuning the actin cytoskeleton.

### n-Butanol production is enhanced by improving the cell growth

**Fig. 5:**
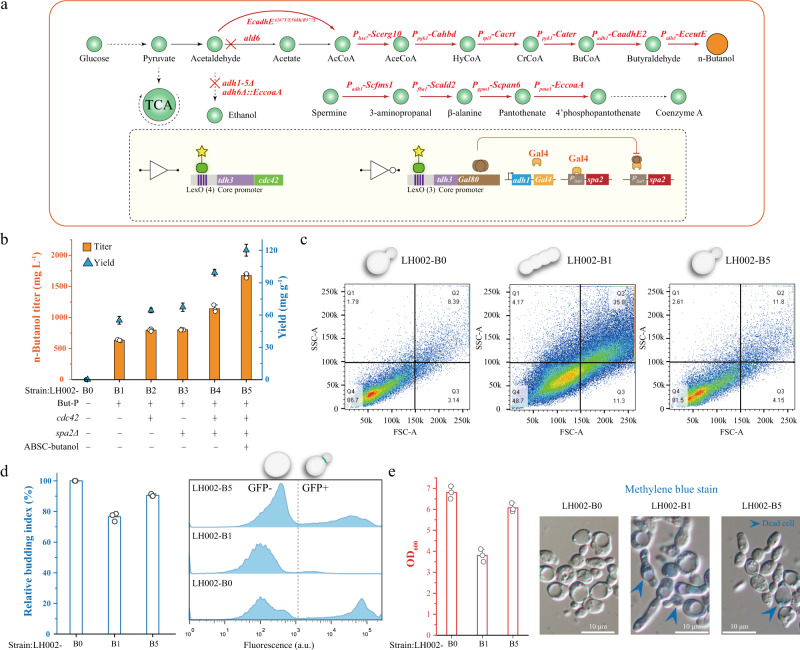
Enhancing n-butanol production by improving cell growth. **a** The schematic diagram of the n-butanol pathway. The red cross symbols indicate that the corresponding gene was knocked out and the red arrows symbols indicate the corresponding gene was overexpressed. But-P means n-butanol pathway. **b** n-Butanol production of different engineered strains in shake flask culture. The histogram graphs were the titer of different engineered strains, whereas the triangle symbols were the yield of the corresponding engineered strain. **c** The population distribution of normal yeast morphology in LH002-B0, LH002-B1, and LH002-B5. The morphology was characterized by SSC v.s. FSC density plot by cell cytometry and 20,000 cells were collected. Each dot or point on the plot represents an individual cell that has passed through the laser. LH002-B0 strain was the control strain without the n-butanol pathway. LH002-B1 strain was only harbored the n-butanol pathway, whereas the LH002-B5 strain was harbored the n-butanol pathway and ABSC-butanol with actin cable genes. **d** The relative cell budding index were analyzed in LH002-B0, LH002-B1, and LH002-B5 strain, respectively. **e** The cell density was characterized by OD_600_. Additionally, methylene blue was applied to stain the dead cells. Three experiments (*n* = 3) were repeated independently with similar results. **b**, **e** Values and error bars represent the mean values and standard deviations of three biological repeats, respectively. Source data are provided as a Source Data file.

*Saccharomyces cerevisiae* LH002-B1 was constructed by inserting genes encoding enzymes related to the n-butanol production pathway from *Clostridium acetobutylicum* (Fig. 5a). Strain LH002-B1 produced 634.3 mg L^−1^ n-butanol (Fig. 5b). Compared with the corresponding values in the control strain LH002-B0, the accumulation of n-butanol in strain LH002-B1 led to (1) elongated cells (Figs. 5c), (2) a 23.5% decrease in the budding index (Figs. 5d), (3) a 44.1% lower of cell density (OD_600_) (Fig. 5e), and (4) a 28.9% decrease in the fraction of viable cells of strain LH002-B1 (Supplementary Fig. 21b). These results indicated that a decreased budding index causes a lower cell growth, thus leading to cell growth defects and potentially limiting the potential for n-butanol production. Therefore, to increase the cell growth, the n-butanol fermentation process was divided into two phases by introducing ABSC-butanol to improve the budding index (Supplementary Fig. 22) as follows: (1) the n-butanol accumulation phase, in which the expression of *spa2* is activated by the *gal1* promoter and the expression of *cdc42* is repressed; (2) the repair phase, in which ABSC-butanol is self-induced by n-butanol, leading to the repression of *spa2* expression by Gal80 and the activation of *cdc42* expression by the *tdh3* promoter.

To autonomously and temporally tune these two phases, ABSC-butanol was introduced into strain LH002-B1, and the DNA sequences that encoded GFP and mKate2 proteins were replaced by *cdc42* and *spa2*, respectively. As shown in Fig. 5b, these manipulations generated a series of strains, namely LH002-B2, LH002-B3, LH002-B4, and LH002-B5. In strain LH002-B5, the expression of *spa2* was activated during the first 50 h of n-butanol fermentation but was repressed thereafter, whereas *cdc42* was increasingly expressed between 50 and 120 h (Supplementary Fig. 23). Based on these manipulations, it was found that: (1) the population distribution of normal yeast morphology was 81.5%, which was 32.8% higher than that of LH002-B1 and 5.2% lower than LH002-B0 (Fig. 5c and Supplementary Fig. 24); (2) the budding index was 14.0% higher than that of strain LH002-B1 and reached 90.6% higher than that of LH002-B0 (Fig. 5d); and (3) the cell density (OD_600_) and the fraction of viable cells was 59.6% and 26.8% higher than that of strain LH002-B1, respectively (Fig. 5e and Supplementary Fig. 21). As a result, the titer, yield, and titer per dry cell weight (DCW) of n-butanol achieved by strain LH002-B5 were 1674.3 mg L^−1^,120.3 mg g^−1^ glucose, and 1511.6 mg g^−1^, respectively (Fig. 5b); these values were substantially higher than those of other strains (titer, yield, and titer per DCW): LH002-B1 (164.0%, 118.2%, and 92.9%, respectively), LH002-B2 (110.0%, 87.0%, and 65.6%, respectively), LH002-B3 (108.3%, 78.8%, and 64.3%, respectively), and LH002-B4 (46.9%, 21.0%, and 21.4%, respectively) (Fig. 5b and Table 1).

**Table 1:** Differences in control strain and engineered *S. cerevisiae* on n-butanol production.

Strain	Characteristics	Titer (mg L^-1^)	Yield (mg g^-1^)	OD_600_	Titer per DCW (mg g^-1^)
LH002-B0	Control strain	0	0	6.8	0
LH002-B1	n-butanol pathway	634.3	55.1	3.8	783.7
LH002-B2	n-butanol pathway+ overexpress of *cdc42*	797.3	64.3	4.1	913.0
LH002-B3	n-butanol pathway+ deletion of *spa2*	803.7	67.3	4.1	920.3
LH002-B4	n-butanol pathway+ overexpress of *cdc42* and deletion of *spa2*	1140.0	99.4	4.3	1244.7
LH002-B5	n-butanol pathway+ABSC-butanol controlled gene expression of *cdc42* and *spa2*	1674.3	120.3	5.2	1511.6

### MCFAs production is enhanced by improving the cell growth

**Fig. 6:**
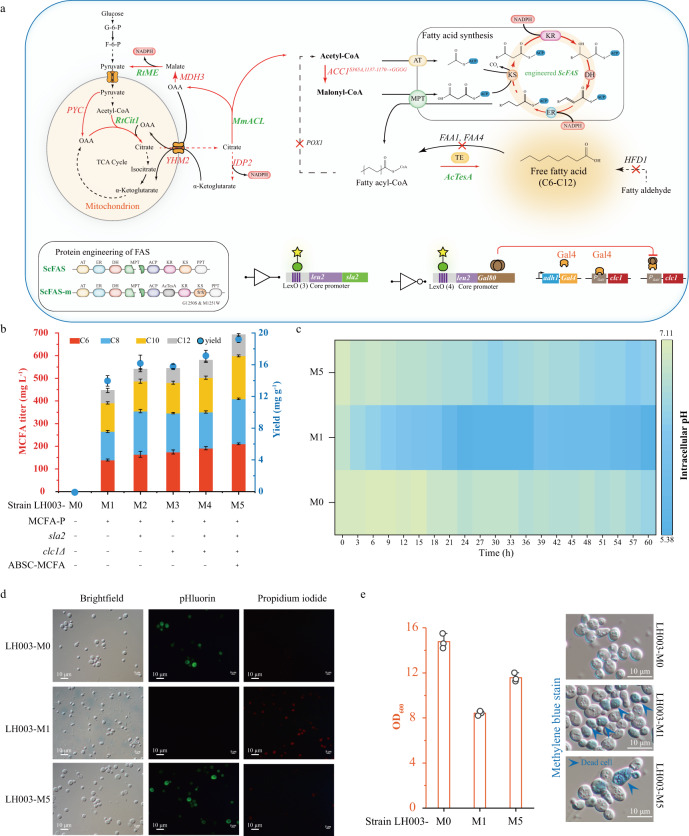
Increasing MCFAs production through increasing cell growth. **a** The schematic diagram of medium-chain fatty acid production. The red arrows indicate the enhancement of carbon flux into medium-chain fatty acids. The thioesterase *Ac*TesA was inserted into fatty acid synthase, and the location of *Ac*TesA was adjacent to the acyl carrier protein. Meanwhile, two mutations (G1250S and M1251W) were performed in Fas2. MCFA-P means MCFAs pathway. **b** MCFA production of different engineered strains in shake flask culture. The histogram graphs were the titer (C6-C12) of different engineered strains, whereas the round symbols were the yield of the corresponding engineered strains. **c** The intracellular pH of strain LH003-M0, LH003-M1, and LH003-M5 during the MCFAs production. LH003-M0 strain was the control strain without the MCFAs pathway and two-phase damage buffer. LH003-M1 strain was only harbored the MCFAs pathway, whereas the LH003-M5 strain was both harbored the MCFAs pathway and the autonomous two-phase damage buffer. **d** The microscopy images of strain LH003-M0, LH003-M1, and LH003-M5 during the MCFAs production. **e** The cell density (OD_600_) was analyzed in LH003-M0, LH003-M1, and LH003-M5 strain, respectively. Additionally, methylene blue was applied to stain the dead cells. **b**, **c**, **e** Values and error bars represent the mean values and standard deviations of three biological repeats, respectively. **d**, **e** Three experiments (*n* = 3) were repeated independently with similar results. Source data are provided as a Source Data file.

*Saccharomyces cerevisiae* LH003-M1 was constructed by reinforcing the endogenous fatty acid pathway and engineering fatty acid synthases (Fig. 6a). Strain LH003-M1 could produce 446.6 mg L^−^1 MCFAs (Fig. 6b). The accumulation of MCFAs in strain LH003-M1 led to a decline in pH_i_ from 7.1 to 5.4, which was 23.9% lower than that of the strain LH003-M0 (Fig. 6c, d) and a 43.0% lower cell density (OD_600_) and a 42.4% decrease in the fraction of viable cells, compared to the corresponding values of the control strain LH003-M0 (Fig. 6e and Supplementary Fig. 25b). These results demonstrate that a lower pH_i_ leads to cell growth defect and ultimately affects MCFAs production in strain LH003-M1. To solve these limitations, the MCFAs fermentation process was divided into two phases by introducing the ABSC-MCFA to stabilize the pH_i_ (Supplementary Fig. 26) as follows: (1) the MCFAs accumulation phase, in which the ABSC-MCFA is inactivated and the genes that encode the actin patch are not manipulated; (2) the repair phase in which the ABSC-MCFA is self-induced by the accumulation of MCFAs, leading to the repression of *clc1* expression by Gal80 and the activation of *sla2* expression by the *tdh3* promoter.

To autonomously and temporally tune these two phases, the ABSC-MCFA was introduced into strain LH003-M1, and the DNA sequences that encoded GFP and mKate2 proteins were replaced by *sla2* and *clc1*, respectively. As shown in Fig. 6b, these manipulations generated a series of strains, namely, LH003-M2, LH003-M3, LH003-M4, and LH003-M5. In strain LH003-M5, the expression of *clc1* under the *gal1* promoter occurred during the first 24 h, whereas the expression of *sla2* was activated between 24 and 60 h (Supplementary Fig. 27). Based on these manipulations, the following phenomena were observed: (1) the pH_i_ was restored to 6.4, which was 18.5% higher than that of strain LH003-M1 and reached 90.1% of the value in LH003-M0 (Fig. 6c, d), and (2) the cell density (OD_600_) and the fraction of viable cells of strain LH003-M5 were 36.0% and 43.8% higher compared to those of strains LH003-M1 and LH003-M0, respectively (Fig. 6e and Supplementary Fig. 25). Consequently, the titer, yield, and titer per DCW of MCFAs produced by strain LH003-M5 were 692.3 mg L^−1^, and 19.3 mg g^−1^ glucose, and 280.2 mg g^−1^, respectively (Fig. 6b). These values were substantially higher compared to those of other strains (titer, yield, and titer per DCW): LH002-M1 (55.1%, 37.3%, and 12.3%, respectively), LH002-M2 (28.1%, 18.7%, and 2.7%, respectively), LH002-M3 (27.2%, 21.5%, and 4.2%, respectively), and LH002-M4 (19.4%, 12.0%, and 1.9%, respectively) (Fig. 6b and Table 2).

**Table 2:** Differences in control strain and engineered *S. cerevisiae* on MCFAs production.

Strain	Characteristics	Titer (mg L^-1^)	Yield (mg g^-1^)	OD_600_	Titer per DCW (mg g^-1^)
LH003-M0	Control strain	0	0	14.8	0
LH003-M1	MCFAs pathway	446.4	14.1	8.4	249.5
LH003-M2	MCFAs pathway + overexpress of *sla2*	540.3	16.3	9.3	272.8
LH003-M3	MCFAs pathway + deletion of *clc1*	544.1	15.9	9.5	268.9
LH003-M4	MCFAs pathway + overexpress of *sla2* and deletion of *clc1*	579.9	17.2	9.9	275.0
LH003-M5	MCFAs pathway + ABSC- MCFA controlled gene expression of *sla2* and *clc1*	692.3	19.3	11.6	280.2

## Discussion

In this study, the actin cytoskeleton was engineered to improve cell growth and enhance the production of n-butanol and MCFAs (Supplementary Fig. 28). For these purposes, three strategies were used: (1) the damage pattern of n-butanol and MCFAs were focused on budding index and pH_i_, respectively; (2) the actin cytoskeleton was engineered to improve the budding index and stabilize the pH_i_; (3) an autonomous bidirectional signal conditioner was constructed to manipulate the gene expression of the actin cytoskeleton upon accumulation of the biofuels. Based on these strategies, the production of n-butanol and MCFAs significantly increased to 1674.3 mg L^−1^ and 692.3 mg L^−1^, respectively. These results demonstrate that engineering the actin cytoskeleton can improve the performance of industrial strains.

As noted above, the biofuels n-butanol and MCFAs could decrease the budding index and pH_i_ of *S. cerevisiae*, respectively. Generally, the plasma membrane is prone to damage by biofuels^37^, owing to their hydrophobicity or lipophilicity. For instance, short-chain alcohols, such as ethanol^16,38^, n-butanol^37^, and isobutanol^39^, can increase the lipid head group space to augment membrane fluidity, reduce membrane thickness, and alter the function of membrane proteins. Additionally, MCFAs can easily insert into the membrane lipid bilayer and perturb membrane composition^40,41^. Moreover, MCFAs can cause membrane damage, allowing toxic compounds to enter the cytoplasm and disrupt physiological functions^6^. To overcome this membrane damage, membrane engineering has been developed by consolidating the microbial barrier, including remodeling the fatty acid chemical structure^42^, modifying the phospholipid head groups^43^, and regulating the membrane functions^44^. As a result, strategies in membrane engineering have been applied to enhance stress tolerance and the production of octanoic acid^43^, ethanol^45^, and n-butanol^46^. Here, we found that n-butanol and MCFAs could damage the budding process and stabilization of pH_i_, respectively. These results provide a comprehensive understanding of the toxic effects of biofuels.

Engineering the actin cytoskeleton could improve the budding index and stabilize the pH_i_ to facilitate *S. cerevisiae* achieve better rates of cell growth. As a critical physiological parameter, the budding index determines cell growth and productivity during chemical production^28^. Strategies to regulate the budding index have focused on the cell division cycle, including engineering cell volume^47,48^, cell senescence^49^, and cell morphology^50^. These strategies allow microorganisms to cope with different environmental stresses, such as product toxicity^49^ and high-temperature stress^51^. However, in this study, the actin cytoskeleton cable was the target for engineering the budding process in *S. cerevisiae*. As a result, the tortuosity of the actin cable was maintained, leading to normal yeast morphology and increased budding index. Compared with engineering the cell division cycle, engineering of the actin cable offers two advantages: (1) direct alleviation of the toxic effect of biofuels and (2) less disruption to the normal cell division cycle. Moreover, pH_i_ is vital for maintaining the enzymatic activity of metabolic enzymes and partly determines cellular homeostasis^52^. Strategies to maintain pHi have focused on engineering weak acid stress response transcription factors^53^ (e.g., War1 and Haa1) and engineering membrane transporters^6^ (e.g., efflux pumps). However, these two strategies might not directly stabilize pH_i_ homeostasis, because toxic compounds may not be exported out of the cells selectively^54^. Here, the actin patch was engineered to improve the endocytic process and ensure correct formation of the vacuole, which is crucial for pH_i_ homeostasis in yeast^55^; consequently, the pH_i_ was normalized in an MCFA producing strain. The engineering of actin patch engineering leads to fewer side effects than engineering transcription factors^39^ and a lower growth burden than engineering transporters to alleviate the pH_i_ defects^22^.

Finally, an ABSC was constructed that could autonomously and temporally regulate the gene expression of the actin cytoskeleton upon the accumulation of biofuels. To construct an efficient microbial cell factory, static strategies (e.g., promoter engineering^56^ and modular engieering^57^) are not sufficient to enable microbial cells to adapt to the stochastic metabolic perturbations and a polytropic fermentation environment^58^. Therefore, autonomous decision-making genetic circuits may be a better solution to adjust microbial cells to intracellular or extracellular changes^59^. These genetic circuits consist of a sensing module, a processing module, and an output module, with the former two modules deciding the utility and efficiency. (i) The sensing module: the biosensor senses the intracellular (e.g., pH_i_^60^, oxygen^61^, substrate^62^, intermediates^63^, and final product^22^) or extracellular signals (e.g., quorum sensing-related molecules^58,64^). A series of genetic circuits have been developed based on these biosensors; a typical genetic circuit depends on a biosensor that detects the level of intermediates in a pathway. For example, a dynamic sensor-regulator system (DSRS), based on the fatty acid-sensing protein FadR, was developed to manage the trade-off between gene expression and fatty acid ethyl ester synthesis^63^. As a result, the DSRS substantially improved the stability of biodiesel-producing strains and increased the titer of fatty acid ethyl ester to 1.5 g L^−1^. Similar to the DSRS, the ABSC system was based on an n-butanol or MCFA responsive promoter; thus, it could autonomously and temporally regulate the gene expression without adding any small-molecule inducers (e.g., IPTG or anhydrotetracycline) and could be used to control the biosynthetic pathway through changing the corresponding product as required. (ii) The processing module: the convertor, such as a nucleic acid- or protein-based state machine^49,65^, Boolean logic gate^66,67^, and CRISPRi system^68^, are key components of the processing module that act to convert the upstream signal into cellular behaviors. In the ABSC system, the endogenous yeast pheromone response pathway was used as the convertor, which could convert signals from biofuel accumulation into endogenous ones to regulate the expression of the corresponding genes. Through this convertor, the ABSC system could maintain cellular homeostasis^69^ while avoiding the problem of expression of genes competing for cellular resources (e.g., energy and carbon building blocks) and providing high compatibility for different conditions.

In conclusion, engineering the actin cytoskeleton provides a platform to improve strain performance during biofuel production and complements the existing strategies that alleviate biofuel’s toxicity (Supplementary Table 1). With the increasing demand for biofuels, it is paramount to design efficient and rational engineering tools to enhance biofuel production based on a comprehensive understanding of the mechanisms for stress tolerance to biofuels. The proposed approach developed here to engineer the cytoskeleton may offer an efficient way to design industrial strains that produce high-value biofuels.

## Methods

### Strains and cultivation conditions

All strains, plasmids, codon-optimized genes, and primers used in this study were listed in Supplementary Data 1–4. The DNA manipulation methods, including ligation cloning procedures (Takara Bio, Dalian, China), one-step cloning kit procedure (Vazyme Biotech Co.,Ltd), Golden gate assembly cloning kit (New England Biolabs, the United States), and CRISPR Cas9-mediated genome engineering procedure^70^ were used for plasmids construction. The guide-RNA used in this study was predicted by the website: https://atum.bio/eCommerce/cas9/input. CRISPR Cas9-mediated genome engineering was applied for gene knockout, gene integrations, and promoter replacement.

For constructing the n-butanol synthesis pathway in *S. cerevisiae* W303-1A, the gene *gpd2* (encoding NAD-dependent glycerol 3-phosphate dehydrogenase) *adh1*, *adh2*, *adh3*, *adh4*, and *adh5* (encoding alcohol dehydrogenase) were knocked out. Then, the *hbd* encoding 3-hydroxybutyryl-CoA dehydrogenase, *crt* encoding 3-hydroxybutyryl-CoA dehydratase, *adhE2* encoding alcohol dehydrogenase, and *ter* encoding trans-enoyl-CoA reductase from *Clostridium acetobutylicum*, and *eutE* encoding butyraldehyde dehydrogenase from *E. coli* were codon-optimized to *S. cerevisiae* and synthesized by Genewiz. After that, these genes consisted of the heterologous n-butanol synthesis cassettes, and then they were integrated into the genomic *ura3-1* locus of *S. cerevisiae* W303-1A. Additionally, *coaA* and *adhE*^*A267T/E568K/R577S*^ from *E. coli* were integrated into genomic *adh6* and *sfa1* locus, respectively. Finally, the native promoter of *fms1*, *erg10*, *ald2*, and *pan6* were replaced into *adh1*, *hxt7*, *fba1*, and *gpm1*, respectively. For n-butanol production in a shake flask, yeast nitrogen base (YNB) medium (6.7 g L^−1^ yeast nitrogen base without amino acids and 20 g L^−^1 glucose) was used as a regular culture for each yeast strain. The seed cultures were inoculated in the YNB medium until reaching the exponential phase. After washing by sterilized double distilled water two times, the seed cultures were inoculated aerobically into a 100 mL flasks with 50 mL fermentation medium^71^ (1.7 g L^−1^ yeast nitrogen base without amino acids, 5 g L^−1^ (NH_4_)_2_SO_4_, 20 mM KH_2_PO_4_, supplemented with 0.171 mM uracil, 0.439 mM leucine, 0.093 mM tryptophan, 0.124 mM histidine, 0.134 mM methionine, 0.08 mM adenine and 20 g L^−1^ glucose) with a starting optical density at 600 nm (OD_600_) of 0.3 for fermenting 120 h under 200 r.p.m at 30 °C.

For constructing MCFA synthesis pathway in *S. cerevisiae* Cen. PK2-1c, the gene pox1 (encoding fatty-acyl coenzyme A oxidase), *faa1*, and *faa4* (encoding long-chain fatty acyl-CoA synthetase) were knockout simultaneously. Then, Fas2-expressing cassettes [*tdh3*_*promoter*_*-Scfas2(G1250S, M1251W, Scacp-‘ActesA)-fba1*_*terminator*_] were integrated into chromosome loci X-2. Moreover, acyl-CoA thioesterase from *Yersinia pestis* (*tesB*), ATP:citrate lyase from *Mus musculus* (*acl*), cytosolic NADP^+^-dependent malic enzyme from *Rhodosporidium toruloides* (*me*) and citrate synthase from *R. toruloides* (*cit*) were were codon-optimized and synthesized by Genewiz. *acl*, *me*, and *cit* expression cassette were integrated into *hfd1* locus, *tef1p-acc1(S659A & 1137-1170* → *GGGG)-adh1t* was integrated into *gpd1* locus, and *tesB* expression cassette was integrated into *eeb1* locus. At last, the native promoter of *pyc1*, *idp2*, and *yhm2* was replaced by *pyk1*, *adh1*, and *gpd1*, respectively. For medium-chain fatty acids (MCFA) production, the pre-cultures were inoculated in modified YPD medium (1% yeast extract, 2% peptone, and 2% dextrose with 200 μg mL^−1^ geneticin and nourseothricin) until reaching the exponential phase under 200 r.p.m at 30 °C. After washing by sterilized double distilled water two times, the seed cultures were inoculated into 250 mL with 50 mL YPD (containing 200 μg mL^−1^ geneticin and nourseothricin) with an initial OD_600_ of 0.1 for fermenting 60 h under 200 r.p.m at 30 °C.

For autonomous and temporal control of actin cytoskeleton in n-butanol and MCFA producing strain, Gibson assembly and molecular cloning techniques were applied to construct the ABSC related plasmids. In n-butanol producing strain, the ABSC system were constructed based on plasmid yEPlac181 (2 μ, Amp^R^, *leu2*) and yCPlac33 (CEN/ARS, Amp^R^, *ura3*). In MCFAs producing strain, to adapt the ABSC system in YPD medium, the auxotrophic marker of yEPlac181 and yCPlac33 were replaced to KanMX4 and NatMX6, and the plasmids were named yEPlac182 and yCPlac34, respectively. Geneticin (200 μg mL^−1^) and nourseothricin (200 μg mL^−1^) were added to maintain the expression of plasmids.

### Measurement of cell growth

For analyzing the spot assay of *S. cerevisiae*, the cells were suspended in YNB medium to OD_600_ of 1, then the cells were diluted in 10-fold dilution gradient, and finally spotted on the YNB medium contained 0.8% n-butanol and 0.2 mM decanoic acid. The cells were incubated at 30 °C for 2 days. For PI stain assay, the cells were incubated to the exponential phase under 200 r.p.m at 30 °C. Then, the cultures were treated with or without the biofuels (n-butanol and decanoic acid) and incubated for another 10 h. After that, the cells were collected, centrifuged, and then resuspended with phosphate-buffered saline (PBS) containing propidium iodide. The cells were dyed at room temperature for 15 min under dark conditions. During the measurement of cell growth, two steps were implemented to prevent evaporation of n-butanol. (1) For analyzing the spot assay, the YNB agar plates contained n-butanol were wrapped with parafilm and taped. (2) For PI stain and cell density analysis, the yeast cells were grown on glass tube with rubber cap and the syringe needle was applied to collect the samples.

Moreover, in this study, one unit of OD_600_ corresponds to a cell dry weight (DCW) of 0.213 g L^−1^. Finally, the cell viability was analyzed by the BD FACSArica III cytometer. And the data were analyzed by FlowJo software (FlowJo-V10).

### Morphological assay of yeast cells

For field emission scanning electron microscope (FESEM) assay, the yeast cells were collected by centrifugation at 5000 *g* for 5 min, and then the precipitate was by phosphate-buffered saline at pH 7.4 for three times. After that, the supernatant was discarded, and then the cells were fixated by adding 2.5% glutaraldehyde at room temperature for 4 h. Later, the cells were washed with phosphate-buffered saline three times. At last, the cells were prepared by the FESEM (FEI Company, Quanta-200). For time-lapse microscopy, yeast cells were cultivated at 30 °C for 8–10 h, then cells were diluted to an optical density OD_600_ = 0.2 in YPD medium, grown for 6 h to mid-log phase. After that, the yeast cells were collected by centrifugation at 5000 g for 5 min, and then the cells were diluted to OD_600_ = 1. Then, 2.5 μL diluted yeast cell culture was placed on a YPD plate in a microscope cavity slide. Finally, the cells were in suit incubated at 30 °C and this time was set as zero-time. Microscopy images were taken using a Nikon ECLIPSE 80i microscope equipped with a ×100 oil-immersion objective.

### Measurement of yeast budding index

To detect the yeast budding index, the yeast cells were incubated on a YNB medium supplemented with needed amino acids for 10 h at 30 °C. Then, the yeast cells were treated with or without 0.8% (v/v) n-butanol treatment for 12 h at 30 °C. After washing by sterilized double distilled water three times, the cell was suspended by YNB medium supplemented with needed amino acids and 20 μM alpha-factor (Sigma-Aldrich, the United States) for synchronizing the cell cycle in the G1 phase. After washing by sterilized double distilled water three times, the treated cells were released into a pre-warmed YNB medium. The released cells were collected every 10 min and then spread on YNB solid plates supplemented with needed amino acid. Lastly, the colony-forming unit (CFU) was calculated to analyze the budding index of each strain. For applying Cdc10-GFP for analyzing relative budding index, the genomic Cdc10 was fused with GFP by using the CRISPR-Cas9 system, and then the cells were observed by fluorescence microscope.

### FM4-64 and alpha-factor labeling assay

For FM4-64 labeling, yeast cells were grown to the mid-log phase, and then cells were stained with FM4-64 for 20 min on ice. After that, cells strain with FM4-64 were released to 30 °C. Finally, the cells were observed at intervals of up to 2 min. For alpha-factor labeling assay, the yeast cells were grown to the mid-log phase and placed on ice for 15 min. After that, the yeast cells were mixed with 2 μM Cy5-α-factor. After 1 h on ice, the yeast cells were washed three times by ice-cold PBS and then resuspended by room temperature PBS to detect the internalization of α-factor by FACS.

### Measurement of cable tortuosity and patch density

Yeast cells were grown to OD_600_ = 0.6 in YPD medium, and then the cells were fixed directly by adding formaldehyde into the medium to a final concentration of 3.7% for 10 min. After that, the cells were centrifuged for 5 min at 5000 g. And, the cells were fixed again by PBS containing 3.7% formaldehyde. After washing by PBS three times, the cells were stained with Alexa Fluor 488-phalloidin at room temperature for 1 h. Finally, the cells were imaged by Leica TCS SP8, and then the ratio of cable length to the distance between cable endpoint (cable tortuosity)^33^ were analyzed by Image J (NIH, http://rsbweb.nih.gov/ij/). For analyzing actin patch density, the genomic Abp1 was fused with GFP and the cells were imaged by Leica TCS SP8, and the cell area and patch amount were analyzed by Image J, respectively.

### pHluorin calibration

The dual excitation of pHluorin was tested by a blue solid-state laser that emits 50 mW of light at 488 nm and a violet diode laser that emits 100 mW of light at 405 nm. In each trial, 20,000 cells were analyzed, and the data were analyzed with FlowJo software. The pHluorin fluorescence was emitted at 510 nm and excited at 410 and 470 nm, respectively. The mean fluorescence intensities of each cell after excitation at 410 nm and 470 nm were recorded and correlated with pHi by using Eqs. 1 and 2^72^.

1 $${{{\mbox{R}}}}_{{{\mbox{i}}}}=\frac{{{{\mbox{Fi}}}}_{410\;{{\mbox{nm}}}}-{{{\mbox{Fi}}}}_{410\;{{\mbox{nm}}} \; {{\mbox{background}}}}}{{{{\mbox{Fi}}}}_{470\;{{\mbox{nm}}}}-{{{\mbox{Fi}}}}_{470\;{{\mbox{nm}}} \; {{\mbox{background}}}}}$$

Where Fi_410 nm_ and Fi_470 nm_ is the mean fluorescence intensities of cells that expressing pHluorin-based pH probes, and Fi_410 nm background_ and Fi_470 nm background_ is mean fluorescence intensities of cells without pHluorin^73^.

2 $${{{{\rm{pH}}}}}={{{\mbox{pK}}}}_{{{\mbox{a}}}}-{{\log }}_{10}\left(\frac{{{{\mbox{R}}}}_{{{\mbox{i}}}}-{{{\mbox{RpH}}}}^{{\max }}}{{{{\mbox{RpH}}}}^{{\min }}-{{{\mbox{R}}}}_{{{\mbox{i}}}}}\right)$$

Where Ri is the result of Eq. (1) that means the emission ratio of a specific pH value, and RpH^max^ and RpH^min^ are the limited value for the ratio at extremely acidic (pH 5.0) and alkaline (pH 8.5), respectively. The constant pK*a* was analyzed by in situ calibration curve^72^.

For analyzing in situ calibration curve, The yeast cells were incubated on the ice for 5 min, then centrifuged and resuspended by ice-cold PBS buffer (1.09 g L^−1^ Na_2_HPO_4_, 0.32 g L^−1^ NaH_2_PO_4_, and 0.9 g L^−1^ NaCl), and then repeat centrifuge-resuspend steps two times. The collected cells were incubated at 37 °C for 30 min with buffered solutions that contained 20 μM nigericin, 150 mM KCl, and 50 mM buffering agents (for pHs ≥ 5.0, sodium acetate; for pHs 6 to 6.75, morpholine-ethane-sulfonic acid; for pHs 7 to 8.5, morpholine-propane-sulfonic acid). After treating the cells with 10 μM nigericin, the cells were incubated on ice for 30 min to equilibrate the intracellular pH with external pH. Then, the mean fluorescence intensities of cells were analyzed through flow cytometry, and plotted the calibration curve between pHi and mean fluorescence intensities. Finally, the pKa was determined from the calibration curve. The measurement of intracellular pH is consistent with previously published studies^6,41,74,75^ in both normal and MCFA treated conditions.

### Mathematical model

GFP or mKate2 expression (Eqs. 3–6) in ABSC-butanol and ABSC-MCFA systems were modeled based on the different concentrations of n-butanol and decanoic acid, respectively. In the computational model, the independent variable is the concentration of n-butanol or decanoic acid, whereas the dependent variable is the fluorescence intensity of mKate2 or GFP. *A*_1_ and *R*_1_ are activation and repression expression of the gene in ABSC-butanol, whereas *A*_2_ and *R*_2_ are the corresponding values in ABSC-MCFA, respectively. B is the concentration of n-butanol, whereas D is the concentration of decanoic acid. All simulations were performed in MATLAB (MathWorks).

3 $${{{\rm{A}}}}_{1}=2806-\frac{2586}{{1+\left(\frac{B}{0.336}\right)^{1.4}}}$$

4 $${{{{{\rm{R}}}}}}_{1}=47+\frac{1424}{1+{\left(\frac{B}{0{{\mbox{.}}}235}\right)}^{2}}$$

5 $${{{\mbox{A}}}}_{2}=4306-\frac{4019}{1+{\left(\frac{D}{0{{\mbox{.}}}368}\right)}^{1{{\mbox{.}}}8}}$$

6 $${{{\mbox{R}}}}_{2}=\frac{1742}{1+{\left(\frac{D}{0{{\mbox{.}}}223}\right)}^{2{{\mbox{.}}}2}}-113$$

### Cell cycle assay

To analyze the cell cycle, the yeast cells were incubated in a YPD medium to OD_600_ of 0.6 at 30 °C. The synthetic alpha-factor (Sigma-Aldrich, the United States) was added into the cultures for synchronizing the cell cycle in the G1 phase, then the mixture was incubated at 30 °C for 2 h. After that, the cells were washed twice by pre-warmed YPD medium, and the synchronized cells were released in pre-warmed YPD medium with or without 0.8 % (v/v) n-butanol. The samples were collected every 15 min, and the cells were then fixed in 70% ethanol for 10 min and kept at 4 °C for further processing. After centrifugation, the pellets were resuspended with ice-cold 50 mM sodium citrate solution containing 0.1 mg mL^−1^ Rnase A and then grown overnight under 30 °C. Finally, after centrifugation, the pellets were suspended with a 50 mM sodium citrate solution containing propidium iodide, and then the mixture was prepared in a FACS tube for analysis by BD FACSArica III cytometer. And the data were analyzed by FlowJo software (FlowJo-V10).

### Flow cytometry assay

The yeast cells were washed twice by PBS buffer and resuspended to an OD600 of 0.1. A BD FACSArica^TM^ III cytometer (BD Biosciences, the United States) was used to measure the fluorescence of each sample. GFP, Annexin V, and NBD-lipids were detected in the FITC channel (488 nm EL, 530/30 nm EF), whereas mKate2 and PI stain were detected in the PE-Cy5-A Red channel (561 nm EL, 670/14 nm EF). 10,000 cells were analyzed for each sample. All data were analyzed by FlowJos software (FlowJo-V10).

### qRT-PCR assay

The yeast cells were cultivated to the mid-log phase and harvested. After washing by DEPC-treated water, total RNA was extracted using the MiniBEST universal RNA extraction kit (Takara Bio, Dalian, China), and cDNA was synthesized by PrimeScript II first-strand cDNA synthesis kit (Takara Bio, Dalian, China). After that, the quantitative analysis of the mRNA level of each sample was performed by using TB Green qPCR Master Mix (Takara Bio, Dalian, China) in Bio-Rad CFX96 Touch (Bio-Rad, the United States). In this study, the *act*1, encoding the *β*-actin, was used as a loading control to normalize the gene expression.

### Fluorescence intensity assay

For measuring the fluorescence intensity, the yeast cells were cultured on a YNB medium supplemented with the needed amino acid for 10 h at 30 °C. The fluorescence of cell culture was measured by a SpectraMax M3 microplate reader (Molecular Devices, the United States). The detection of GFP fluorescence intensity was set at 480 ± 10 nm of excitation wavelength and 515 ± 10 nm of emission wavelength. And the detection of mKate2 fluorescence intensity was set at 588 ± 10 nm of excitation wavelength and 645 ± 10 nm of emission wavelength.

### Metabolite quantification

For n-butanol quantification, the fermentation broth was centrifuged at 8000 g for 10 min, and the supernatant was collected. Then the n-butanol concentration was determined by gas chromatography-mass spectroscopy (GC-MS) an Agilent 7890 A GC system and HP-INNOWAX column. For the extraction of medium-chain fatty acids (MCFAs), the fermentation broth was centrifuged at 8000 g for 10 min, and the supernatant was collected. Then, the mixture of hexane: chloroform (4:1, vol/vol) was used for extracting the MCFAs. After that, the organic layer was collected and evaporated through nitrogen. To measure the concentration of MCFAs, samples were resolved in a mixture of methanol: sulfuric acid: chloroform (30:3:1, vol/vol/vol) to heat at 70 °C for 1 h. Subsequently, the Fatty acid methyl esters (FAMEs) were extracted by hexane. Finally, the FA methyl esters were measured GC-MS on an Agilent 7890 A GC system and a Rxi-5Sil column (0.25 mm internal diameter 0.10 μm film thickness, 30 m length), following the method of an initial temperature of 35 °C held for 1 min, 6 °C/min to 200 °C, 30 °C/min to 270 °C, held for 1 min.

### Statistical and reproducibility

The significance of groups of data was determined by a two-tailed Student’s *t*-test by SPSS statistics software (SPSS R24.0.0.0). The data analysis and graphing were performed by Origin 2019 64 bit. All experiments in this study were performed in three biological repeats.

### Reporting summary

Further information on research design is available in the Nature Research Reporting Summary linked to this article.

## Supplementary information

Supplementary Information

Description of Additional Supplementary Files

Supplementary Data 1

Supplementary Data 2

Supplementary Data 3

Supplementary Data 4

Reporting Summary

## Data Availability

Data supporting the findings of this work are available within the paper and its Supplementary Information files. A reporting summary for this article is available as a Supplementary Information file. Source data are provided with this paper, which is also available at Figshare [10.6084/m9.figshare.19329092.v1]. Source data are provided with this paper.

## Source data

Source Data
