# Supplementary material for: Enhancing biofuels production by engineering the actin cytoskeleton in Saccharomyces cerevisiae
Source: Nat Commun. 2022 Apr 7;13:1886. doi: 10.1038/s41467-022-29560-6 (PMC8991263; doi:10.1038/s41467-022-29560-6)
Supplement: Supplementary file 1 — Supplementary Information [file 41467_2022_29560_MOESM1_ESM.pdf]

# Enhancing biofuels production by engineering actin cytoskeleton in *Saccharomyces cerevisiae*

Liu *et al*.

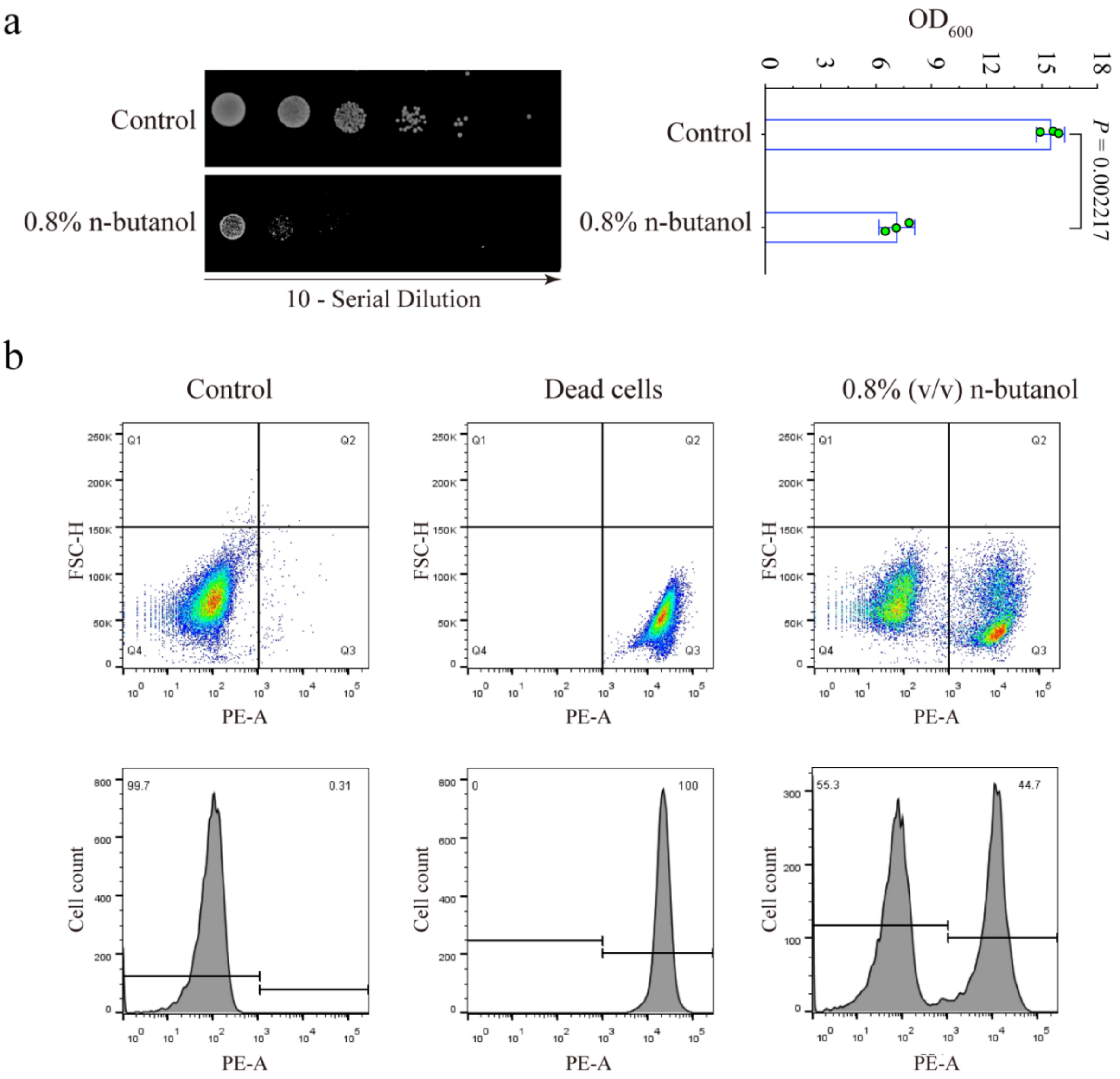


**Supplementary Figure 1. Effect of n-butanol on cell growth. a**, The spot assay and cell density ($OD_{600}$) were analyzed in the control and 0.8% (v/v) n-butanol treated group. Three experiments (n= 3) were repeated independently with similar results. **b**, propidium iodide (PI) stain was applied to analyze the distribution of cell viability in the control and n-butanol treated group. A gate was designed based on forward and side scatter (>99% cells were chosen for the analysis of fluorescence density percentage). All data were exported in FCS3 format and processed using Flow Jo software (FlowJo-V10). *P* values are from a Student's two-sided t-test of the difference from the control group (0% n-butanol). Source data are provided as a Source Data file.

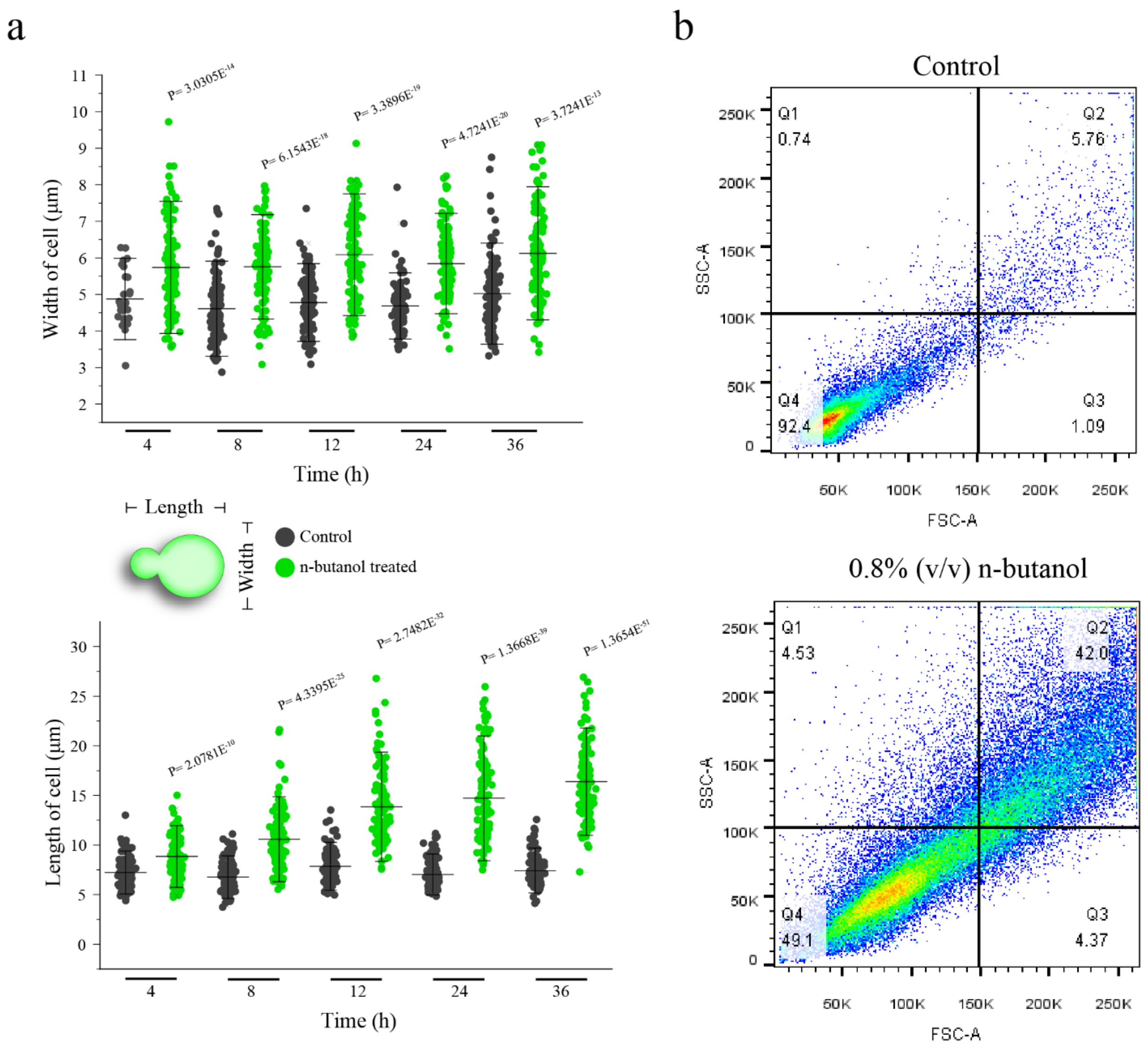


**Supplementary Figure 2. Effect of n-butanol on yeast cell size. a**, Cell width and cell length were detected by the microscope, and 120 cells were analyzed both in the control and n-butanol treated group. Values are shown as mean ± S.D. from 120 (n = 120) cells over three independent biological replicates. **b**, the effect of n-butanol on morphology in *S. cerevisiae*, which was characterized by SSC v.s. FSC density plot by cell cytometry and 20,000 cells were collected. Each dot or point on the plot represents an individual cell that has passed through the laser. *P* values are from a Student's two-sided t-test of the difference from the untreated control group. Source data are provided as a Source Data file.

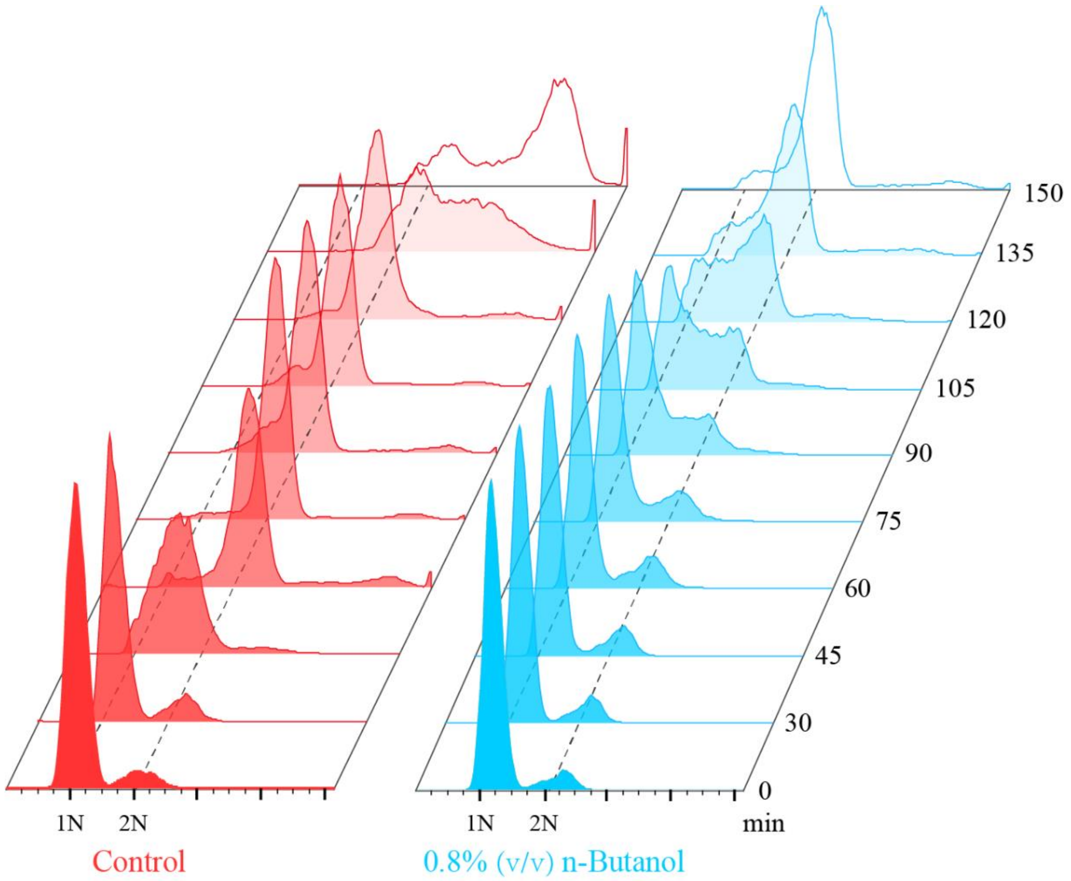


**Supplementary Figure 3. Flow cytometry was used to analyze the cell cycle of control and 0.8% (v/v) n-butanol treated cells.** The cells were synchronized by alpha-factor, and the cells were released into a pre-warmed YPD medium, and the DNA content was analyzed by FACS every 15 minutes. Source data are provided as a Source Data file.

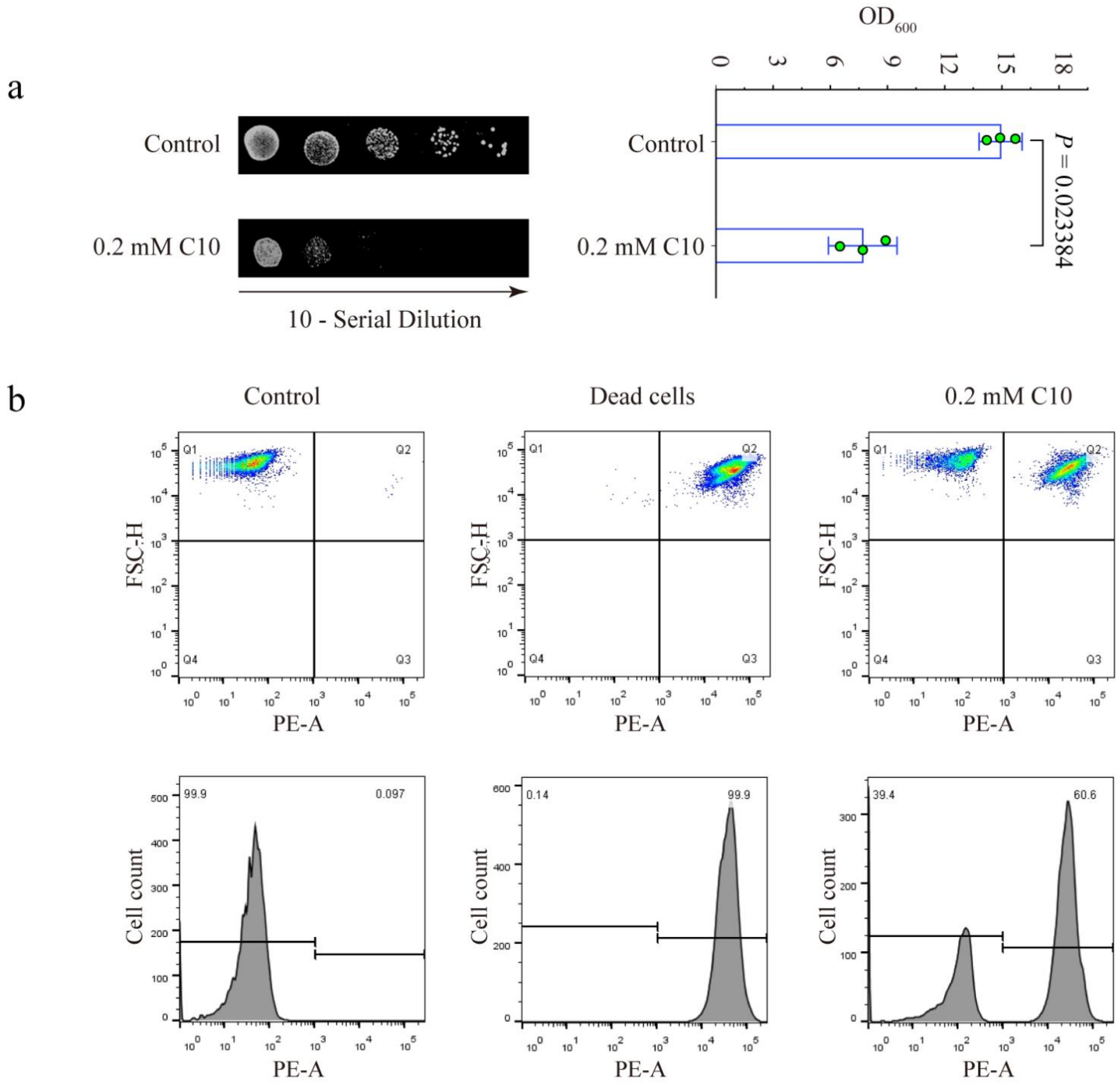


**Supplementary Figure 4**. **Effect of decanoic acid (C10) on cell growth. a**, The spot assay and cell density ($OD_{600}$) were analyzed in the control and 0.2 mM decanoic acid treated group. Three experiments (n= 3) were repeated independently with similar results. **b**, propidium iodide (PI) stain was applied to analyze the distribution of cell viability in the control and decanoic acid treated groups. *P* values are from a Student's two-sided t-test of the difference from the control group (0 mM C10). Source data are provided as a Source Data file.

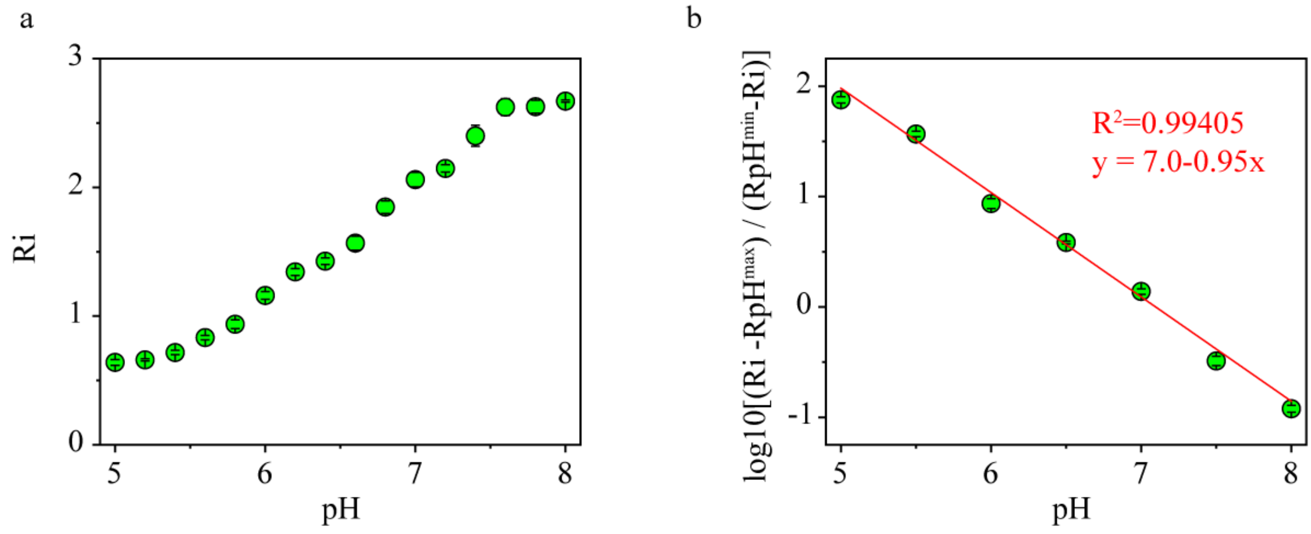


**Supplementary Figure 5. The calibration curve of pHluorin. a**, The calibration curve of pHluorin presented as a plot of the fluorescence excitation ratio versus. The ratio (R) between the emission intensities after excitation at excitation maxima of 410 and 470 nm were calculated using equation 1. Values are shown as mean ± S.D. from three (n = 3) biological replicates. **b**, The calibration curve of pHluorin presented as a logarithmic plot of the fluorescence excitation ratio (a) versus pH. The pHluorin $pK_a$ of 7.0 was calculated from a calibration curve. Values are shown as mean ± S.D. from three (n = 3) biological replicates. Source data are provided as a Source Data file.

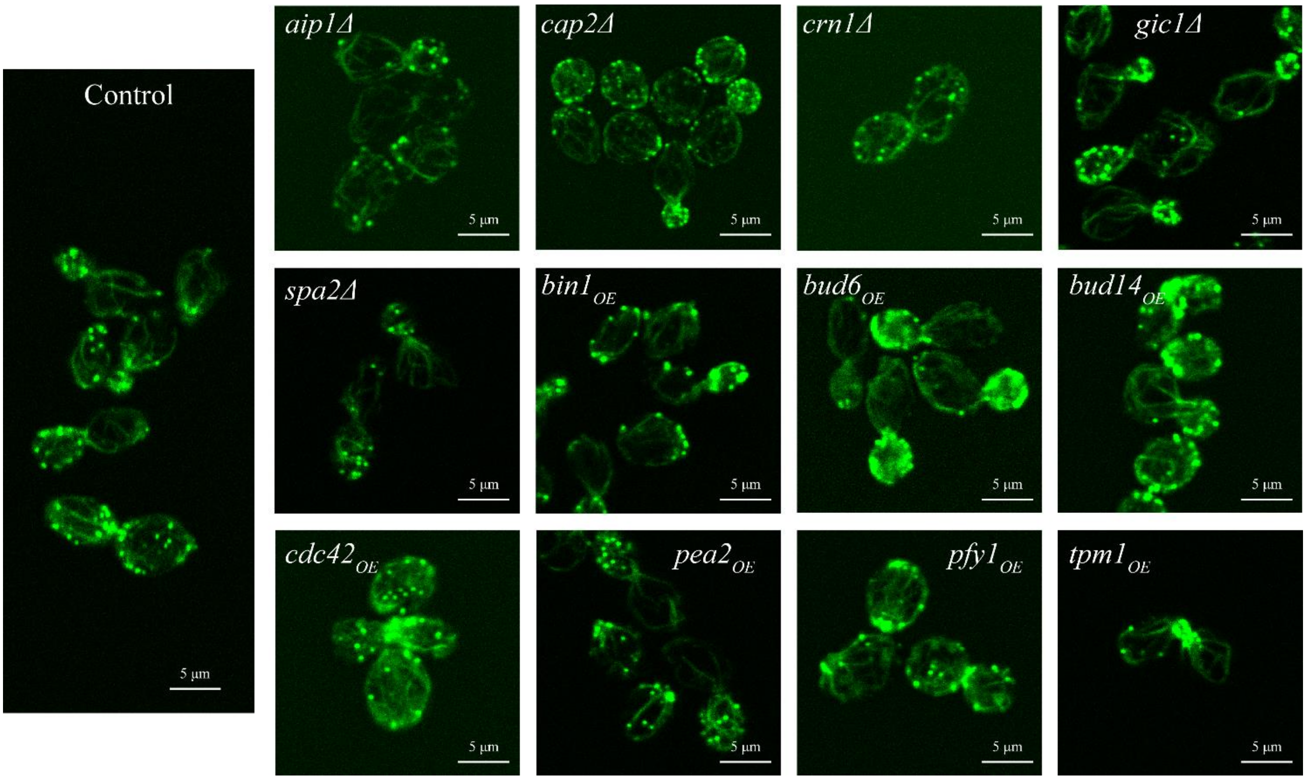


**Supplementary Figure 6. The cells were fixed by formaldehyde and then stained by FITC-phalloidin.** The tortuosity is defined as the ratio of the cable length (*l*) to the distance (*d*) between its two endpoints. 12 candidates were observed by fluorescence microscope, and then the tortuosity was analyzed by Image J. The subscript OE means overexpression. Three experiments (n= 3) were repeated independently with similar results. Source data are provided as a Source Data file.

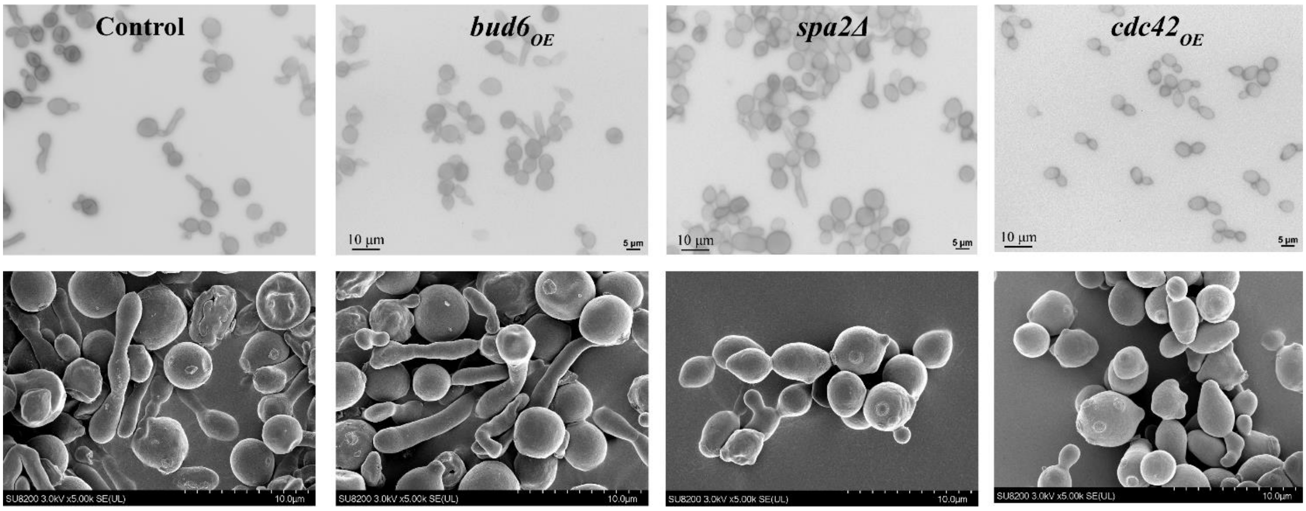


**Supplementary Figure 7**. **Representative cell images of morphology in control strain, *SPA2Δ* strain, *BUD6* overexpression strain, and *CDC42* overexpression strain under 0.8% (v/v) n-butanol treatment.** The cells were stained by calcofluor white. Image colors were inverted. In addition, the morphological change of engineered strain was confirmed by field emission scanning electron microscopy (FESEM). The subscript OE means overexpression. Three experiments (n= 3) were repeated independently with similar results. Source data are provided as a Source Data file.

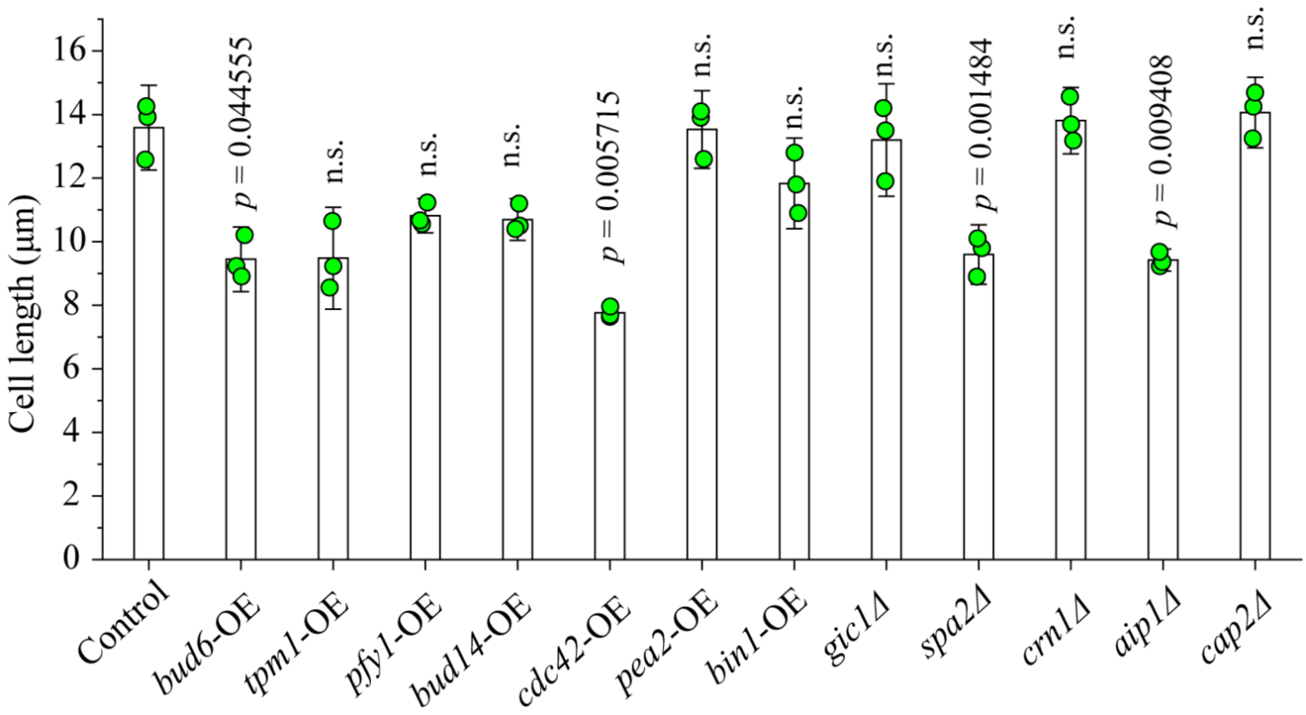


**Supplementary Figure 8. Cell length of individual overexpression or deletion strains under 0.8% (v/v) n-butanol treatment.** Statistical significance were analyzed between control group and different actin patch engineered strains. Significance (*p*-value) was evaluated by two-sided t-test. Values and error bars represent the mean values and standard deviations of three biological repeats, respectively. n.s., not significant. Source data are provided as a Source Data file.

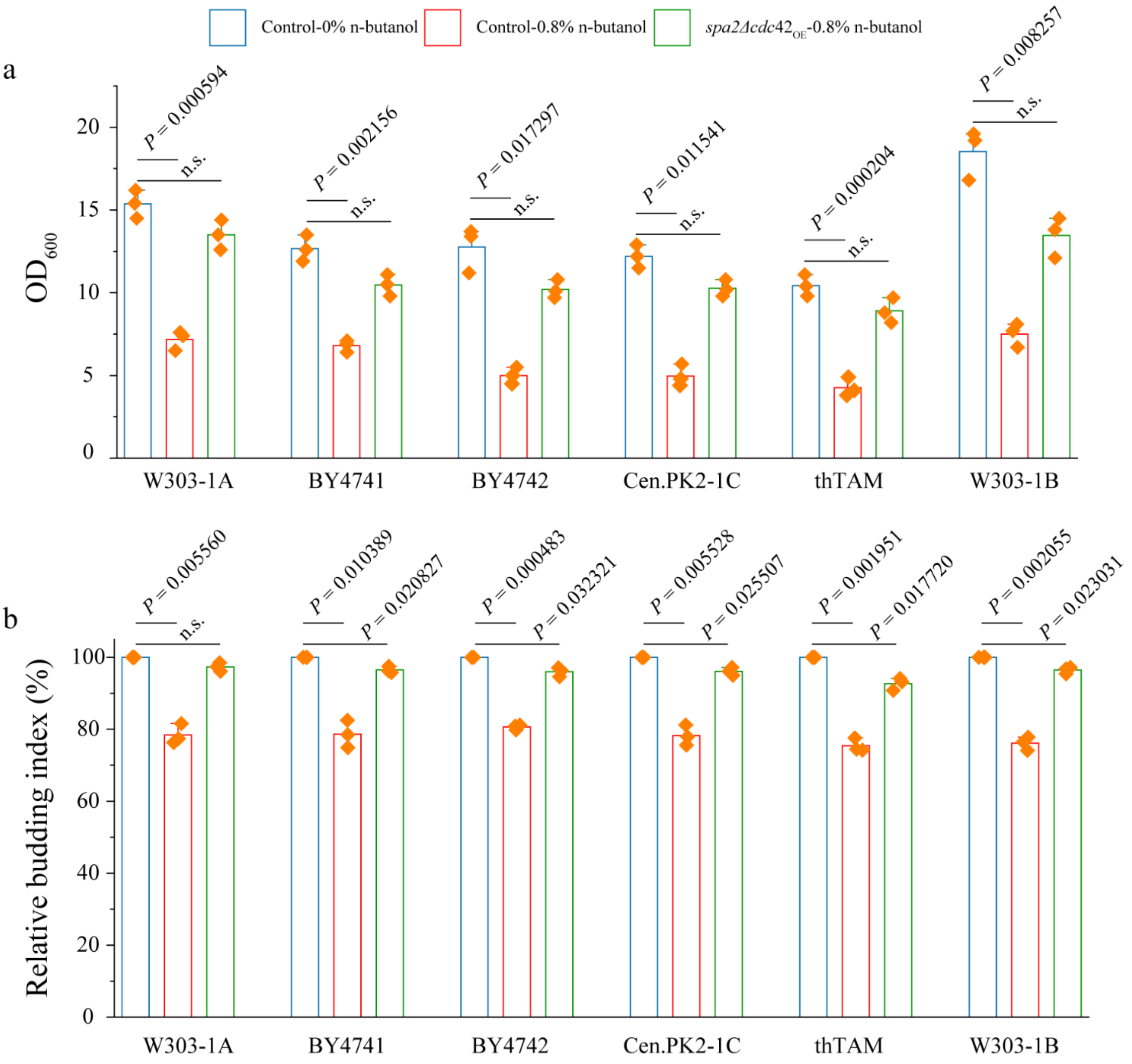


**Supplementary Figure 9. Effect of n-butanol on cell growth in different engineered strains and corresponding engineered strains with *SPA2Δ* and *CDC42* overexpression. a**. The cell density ($OD_{600}$) and **b**. relative budding index of strain W303-1A, W303-1B, BY4741, BY4742, Cen.Pk2-1C, and thTAM under n-butanol treatment. *P* values are from a Student's two-sided t-test of the difference from the control group (0% n-butanol). n.s., not significant. Values in Supplementary Fig. 9 are shown as mean ± S.D. from three (n = 3) biological replicates. Source data are provided as a Source Data file.

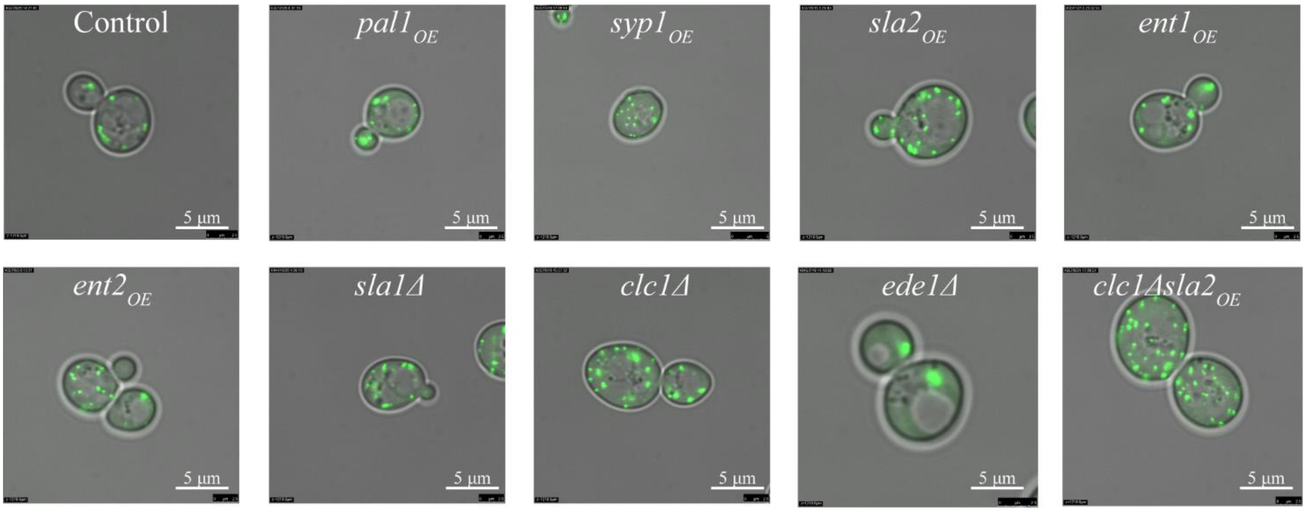


**Supplementary Figure 10. The fluorescence microscope images of the actin patch.** The density of the patch determined the endocytosis process of *S. cerevisiae*. The effect of improving the density of the patch was analyzed by fluorescence microscope and the GFP dot indicated the actin patch. The patch was characterized by fusing the GFP with the genomic Abp1. Three experiments (n= 3) were repeated independently with similar results. Source data are provided as a Source Data file.

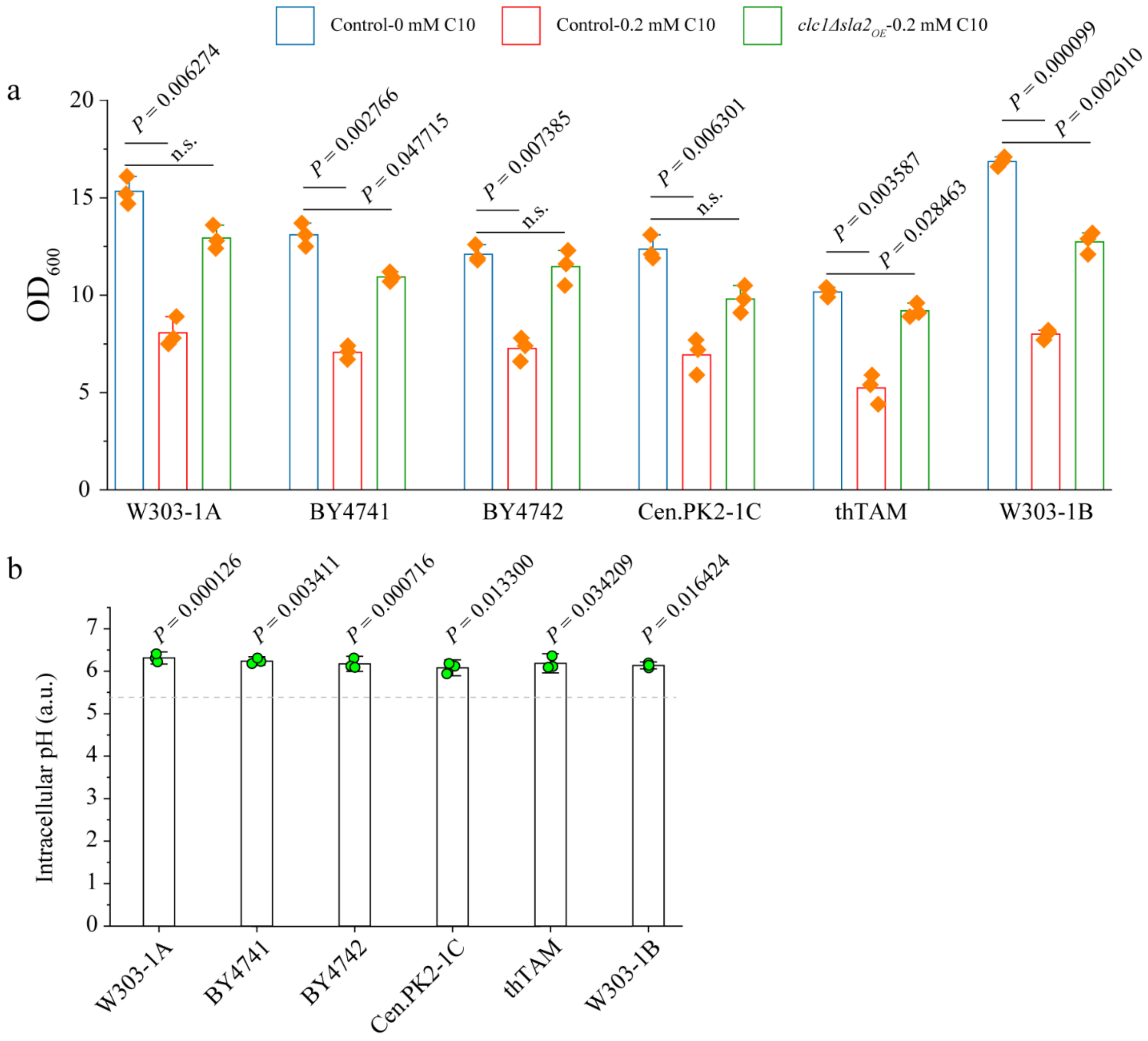


**Supplementary Figure 11. Effect of decanoic acid on cell growth and intracellular pH in different strains and corresponding engineered strains with *CLCΔ* and *SLA2* overexpression. a.** The cell density ($OD_{600}$).and **b**. $pH_i$ of strain W303-1A, W303-1B, BY4741, BY4742, Cen.Pk2-1C, and thTAM under decanoic acid (C10) treatment. The grey dashed line was the intracellular pH of control strain under 0.2 mM C10 treatment. Significance (*p*-value) was evaluated by two-sided t-test. n.s., not significant. Values in Supplementary Fig. 11 are shown as mean ± S.D. from three (n = 3) biological replicates. Source data are provided as a Source Data file.

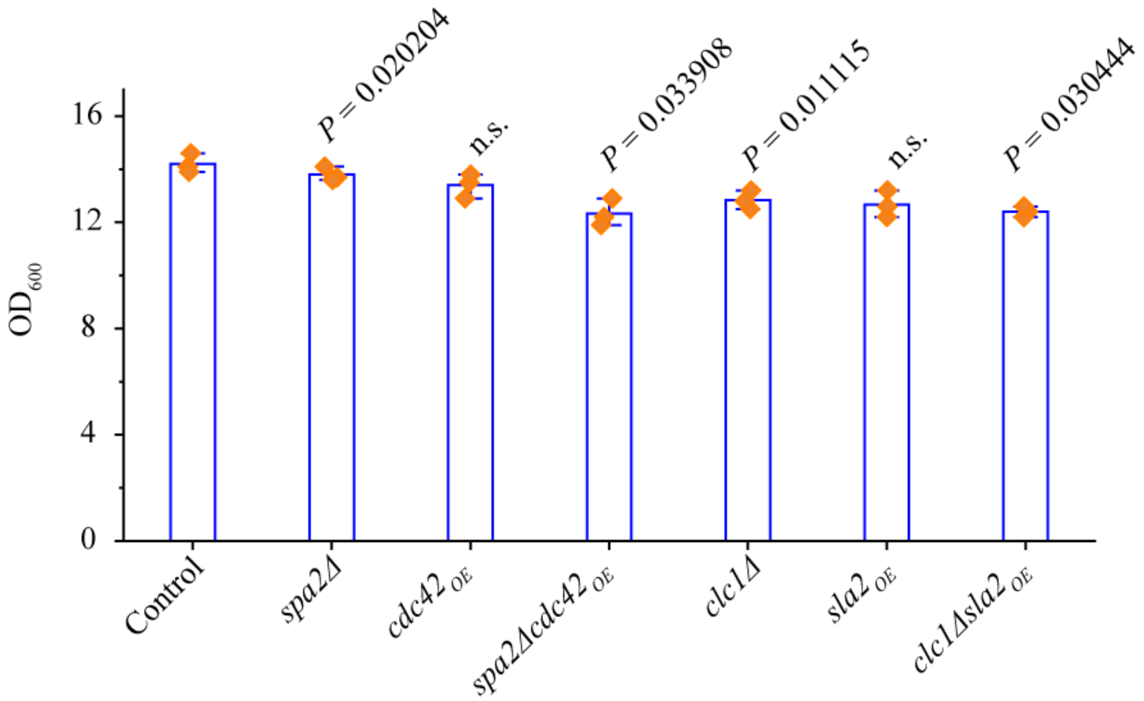


**Supplementary Figure 12. The $OD_{600}$ of control strain, *spa2Δ* strain, *cdc42* overexpression strain, *spa2Δcdc42*$_{OE}$ stain, *clc1Δ* strain, *sla2* overexpression strain, and *clc1Δsla2*$_{OE}$ strain on YNB medium were analyzed, respectively.** *P* values are from a Student's two-sided t-test of the difference from the control group. n.s., not significant. Values are shown as mean ± S.D. from three (n = 3) biological replicates. Source data are provided as a Source Data file.

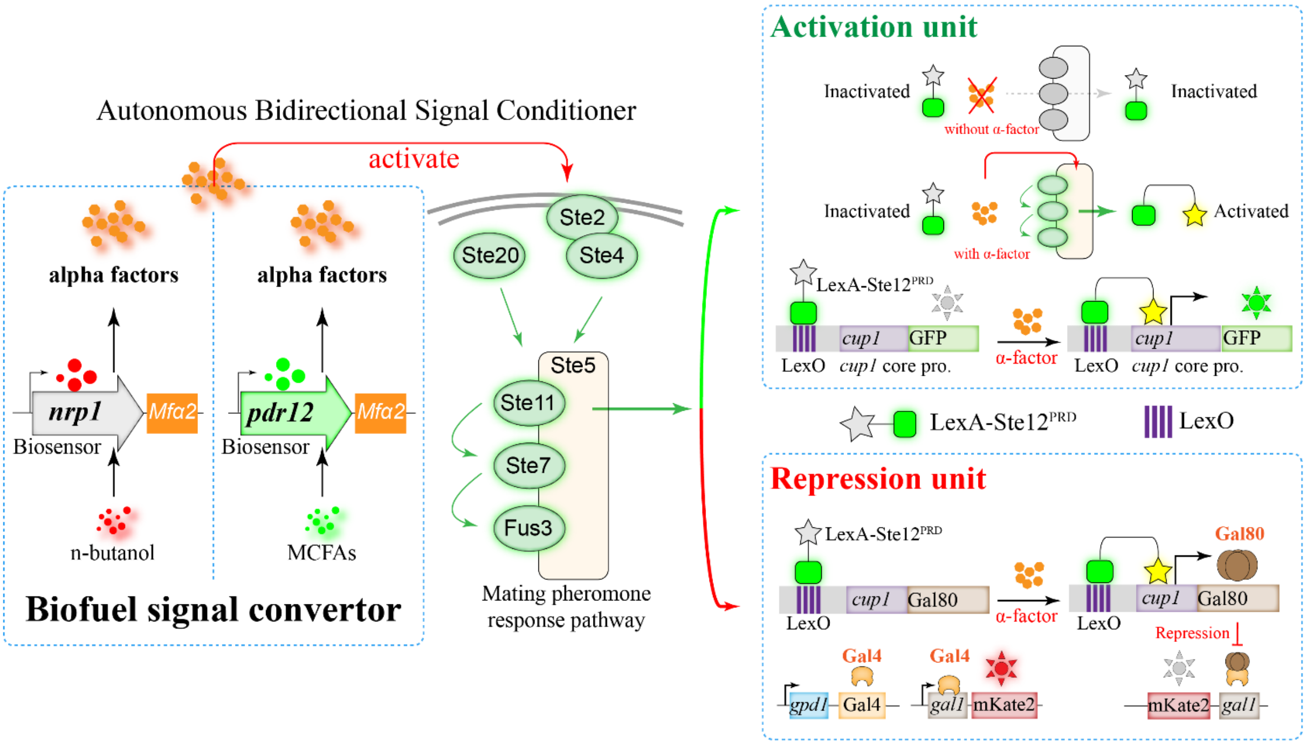


**Supplementary Figure 13. The schematic diagram of the autonomous bidirectional signal conditioner (ABSC).**

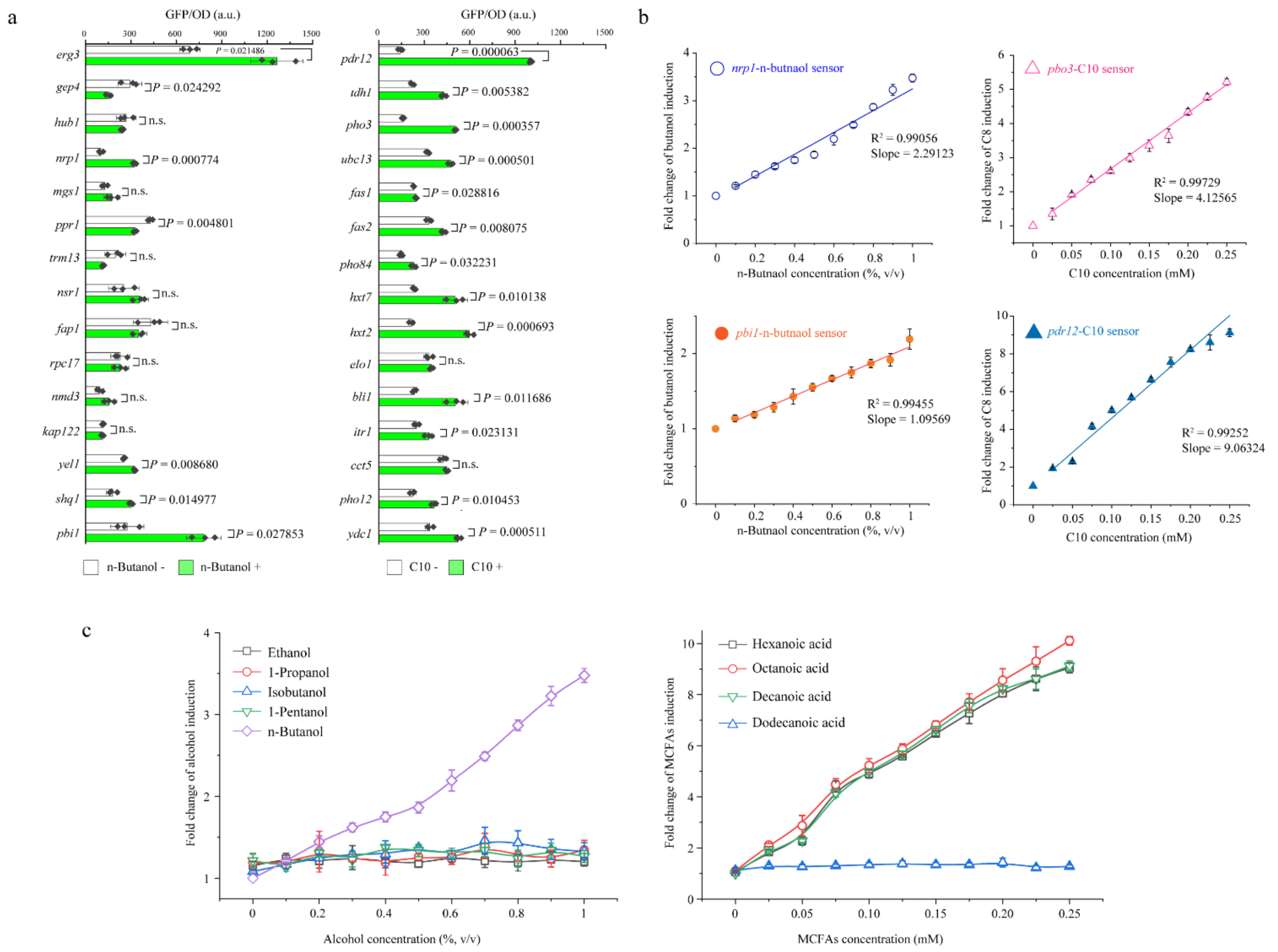


**Supplementary Figure 14. Screening the biofuels responsive promoters. a**, The first round to screen the biofuels responsive promoters in n-butanol and decanoic acid condition, respectively. Values are shown as mean ± S.D. from three (n = 3) biological replicates. **b**, The potential biofuels responsive promoters were characterized in a series level of n-butanol or decanoic acid, respectively. **c**, The specificity of *NRP1* and *PDR12* to different analogous compounds. Abbreviation: C10, decanoic acid. *P* values are from a Student's two-sided t-test of the difference from the control group. n.s., not significant. Values are shown as mean ± S.D. from three (n = 3) biological replicates. Source data are provided as a Source Data file.

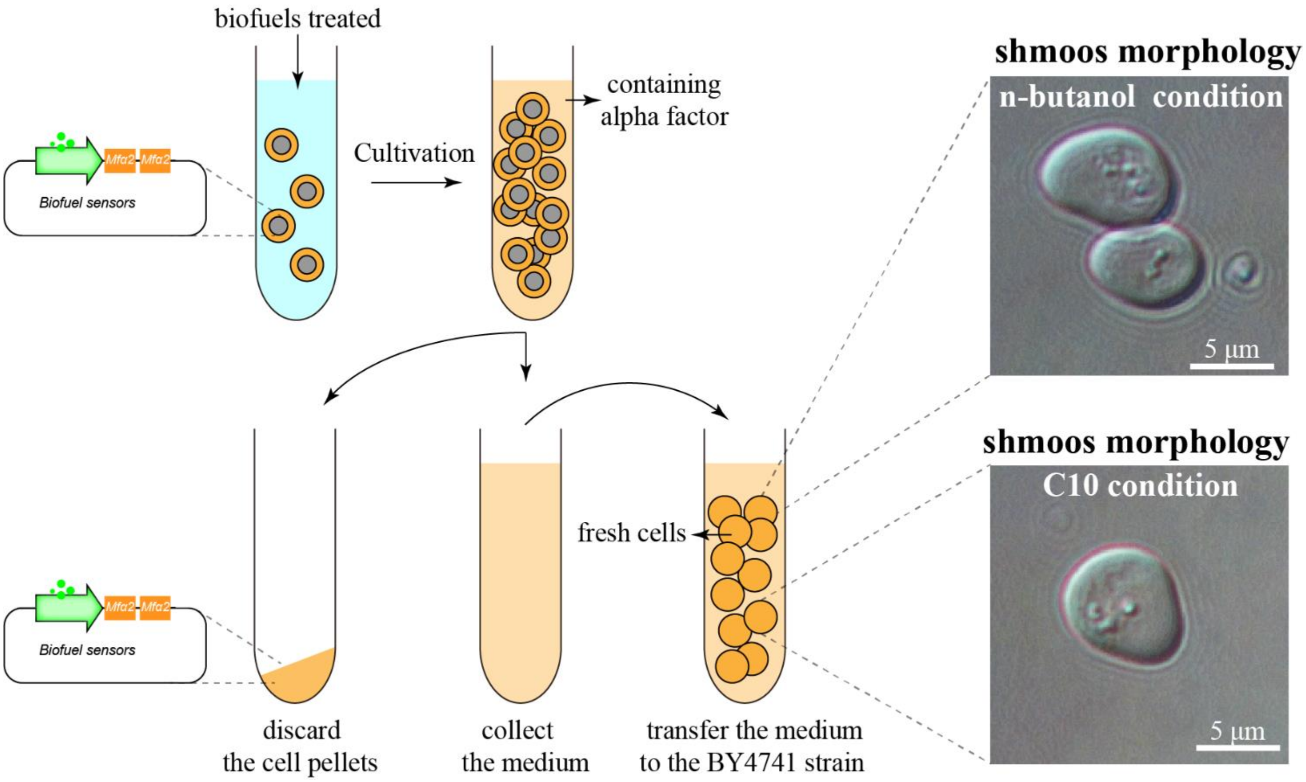


**Supplementary Figure 15. Characterization of the validity of biofuel signal convertor.** The expression of alpha-factor was regulated by biofuels responsive promoters, which were activated under biofuels (n-butanol and medium-chain fatty acid) treatment. After 8 h treatment in 0.2 mM MCFA (C10) or 12 h in 0.8% (v/v) n-butanol, the medium was collected, and then the *S. cerevisiae* BY4741 was cultivated by the collected medium. Finally, the *S. cerevisiae* BY4741 cell showed shmoos morphology, indicating that the biofuel signal converter could convert the biofuels' signal into endogenous signals. Three experiments (n= 3) were repeated independently with similar results. Source data are provided as a Source Data file.

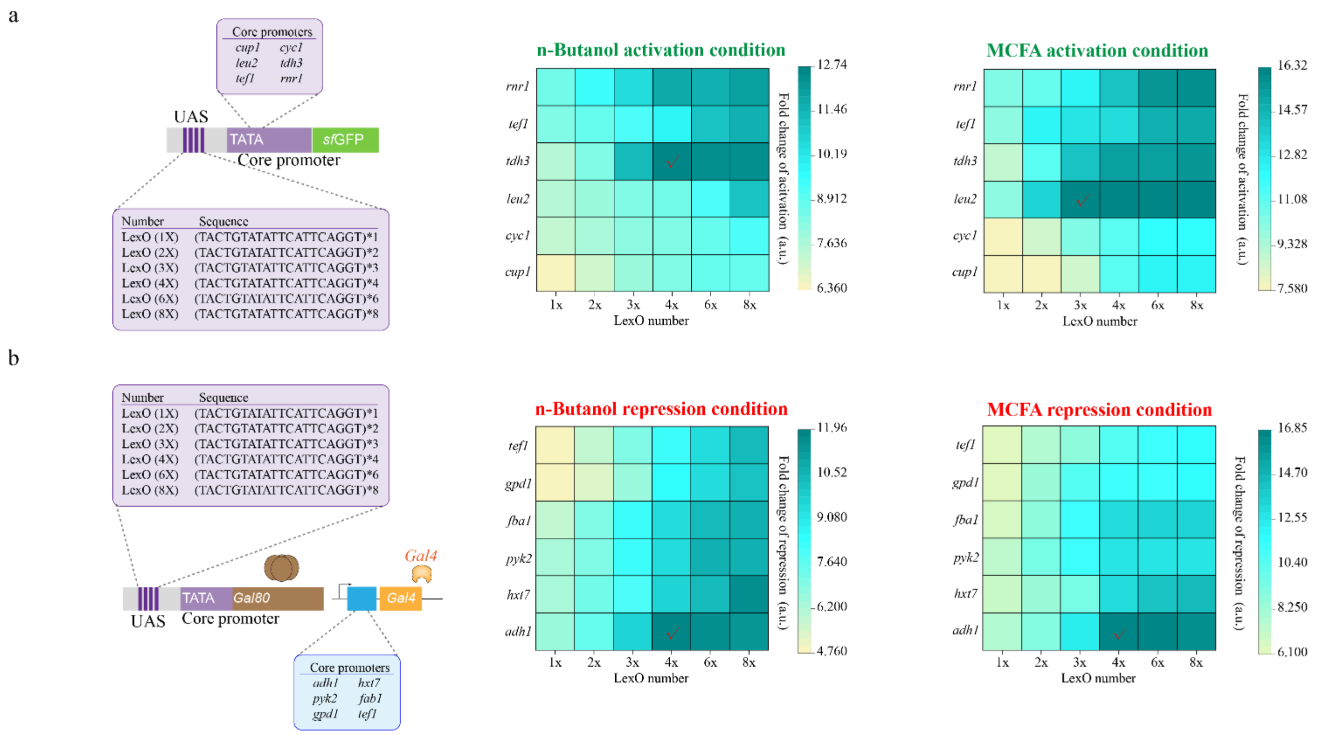


**Supplementary Figure 16. Optimization and characterizing ABSC. a**, The optimization of the activation unit. To obtain a more efficient ABSC, altering the number of LexO that modulate the binding of LexA-Ste12$^{PRD}$and changing the identity of the core region of the promoters that alter the rate of transcriptional initiation has been applied. **b**, The optimization of the activation unit. The number of LexO and promoters that activated the expression of Gal4 had been optimized to expand the dynamic range of ABSC. Source data are provided as a Source Data file.

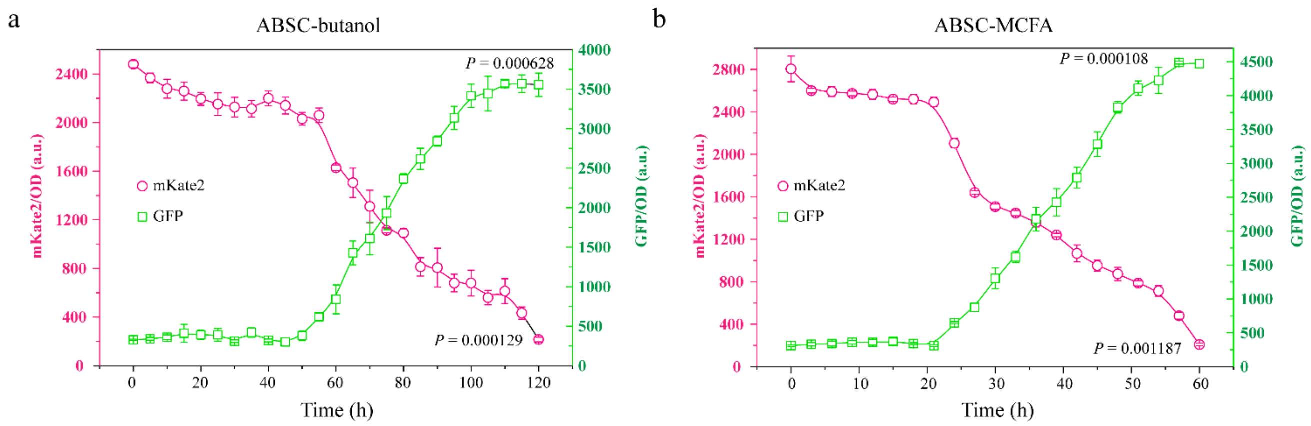


**Supplementary Figure 17. Regulation mode of ABSC systems in n-butanol and medium-chain fatty acids conditions. a-b**, The regulation mode of ABSC-butanol and ABSC-MCFA in fed-batch level (5-L bioreactors). *P* values are from a Student's two-sided t-test of the difference between 120 h (or 60 h) and 0 h. Values in Supplementary Fig. 17 are shown as mean ± S.D. from three (n = 3) biological replicates. Source data are provided as a Source Data file.

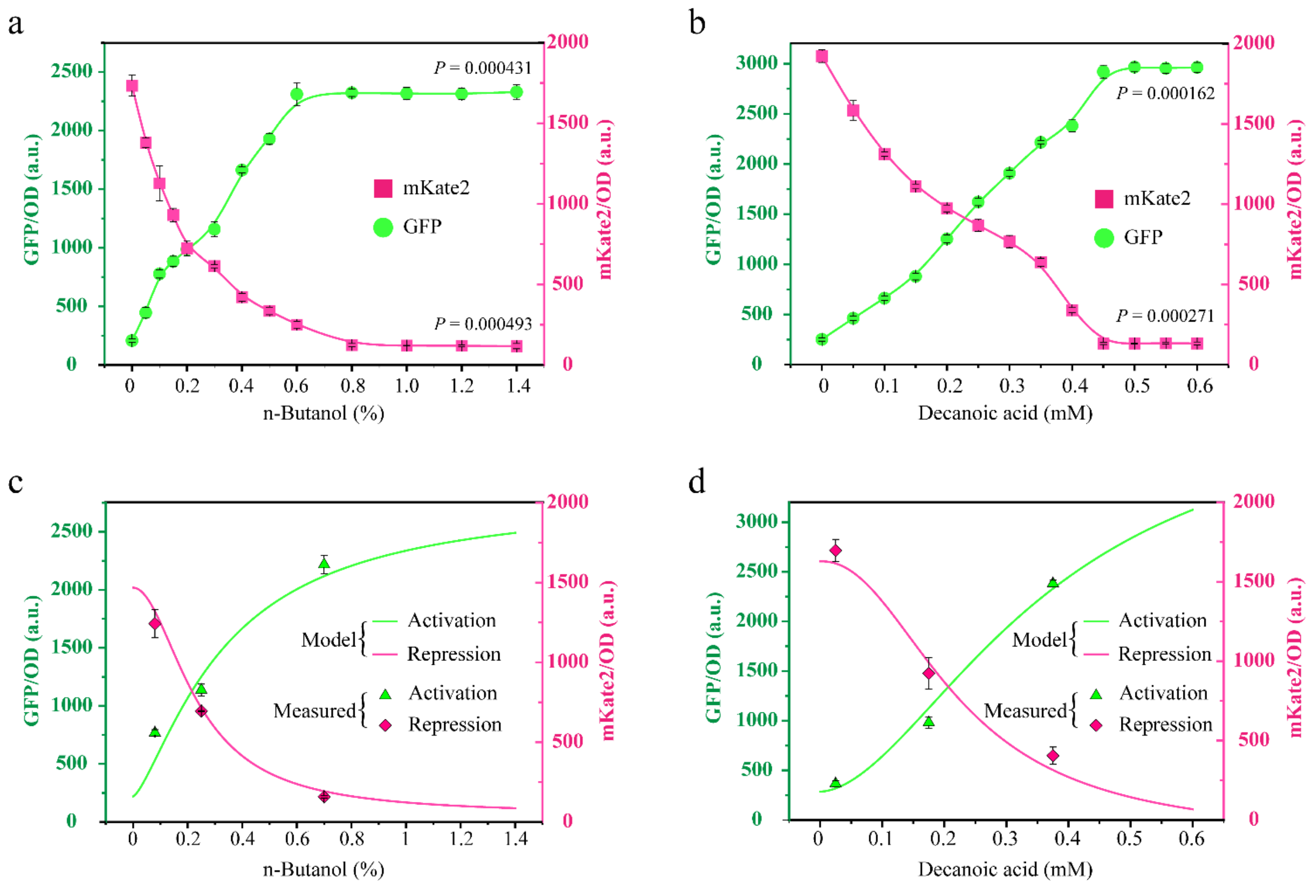


**Supplementary Figure 18. Mathematical modeling of ABSC system in n-butanol and decanoic acid conditions. a-b**, The dual control of ABSC system was characterized. The synthetic promoter LexO-*TDH3*/*LEU2* can both activate the expression of GFP and Gal80 (a repressor of *GAL1* promoter) in butanol and decanoic acid, respectively. **c-d**, Mathematical modeling predictions for ABSC-butanol and ABSC-MCFA system, respectively. In the mathematical model, the independent variable is the concentration of n-butanol or decanoic acid, whereas the dependent variable is the fluorescence intensity of mKate2 or GFP, respectively. Additionally, the experimental data were used to validated the accuracy of these models by growing cells in the presence of three different concentration of n-butanol (0.08%, 0.25%, and 0.7%) and decanoic acid (0.025 mM, 0.175 mM, and 0.375 mM) and then measuring the expression levels of GFP and mKate2, respectively. *P* values are from a Student's two-sided t-test of the difference between 1.4% and 0% n-butanol (or 0 mM and 0.6 mM C10). Values in Fig.S18 are shown as mean ± S.D. from three (n = 3) biological replicates. Source data are provided as a Source Data file.

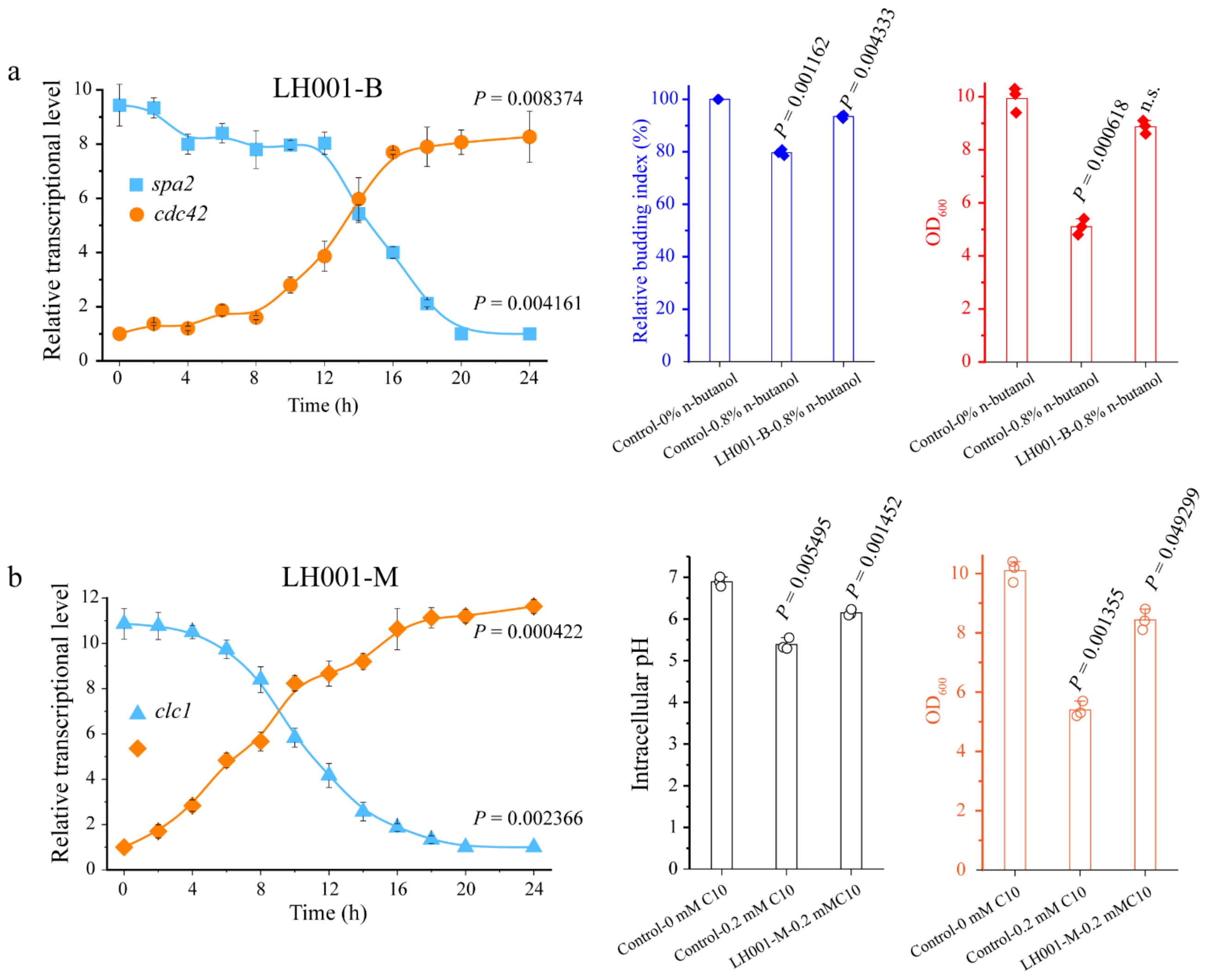


**Supplementary Figure 19**. **The regulation mode of ABSC system on endogenous genes. a**, The relative expression of *spa2* and *cdc42* in strain LH001-B. The relative budding index and $OD_{600}$ of strain LH001-B compared with that of the control strain. **b**, The relative expression of clc1 and sla2 in strain LH001-M. The intracellular pH and $OD_{600}$ of strain LH001-M compared with that of the control strain. a-b, Values and error bars represent the mean values and standard deviations of three biological repeats, respectively. Significance (*p*-value) was evaluated by Student's two-sided t-test. n.s., not significant. Source data are provided as a Source Data file.

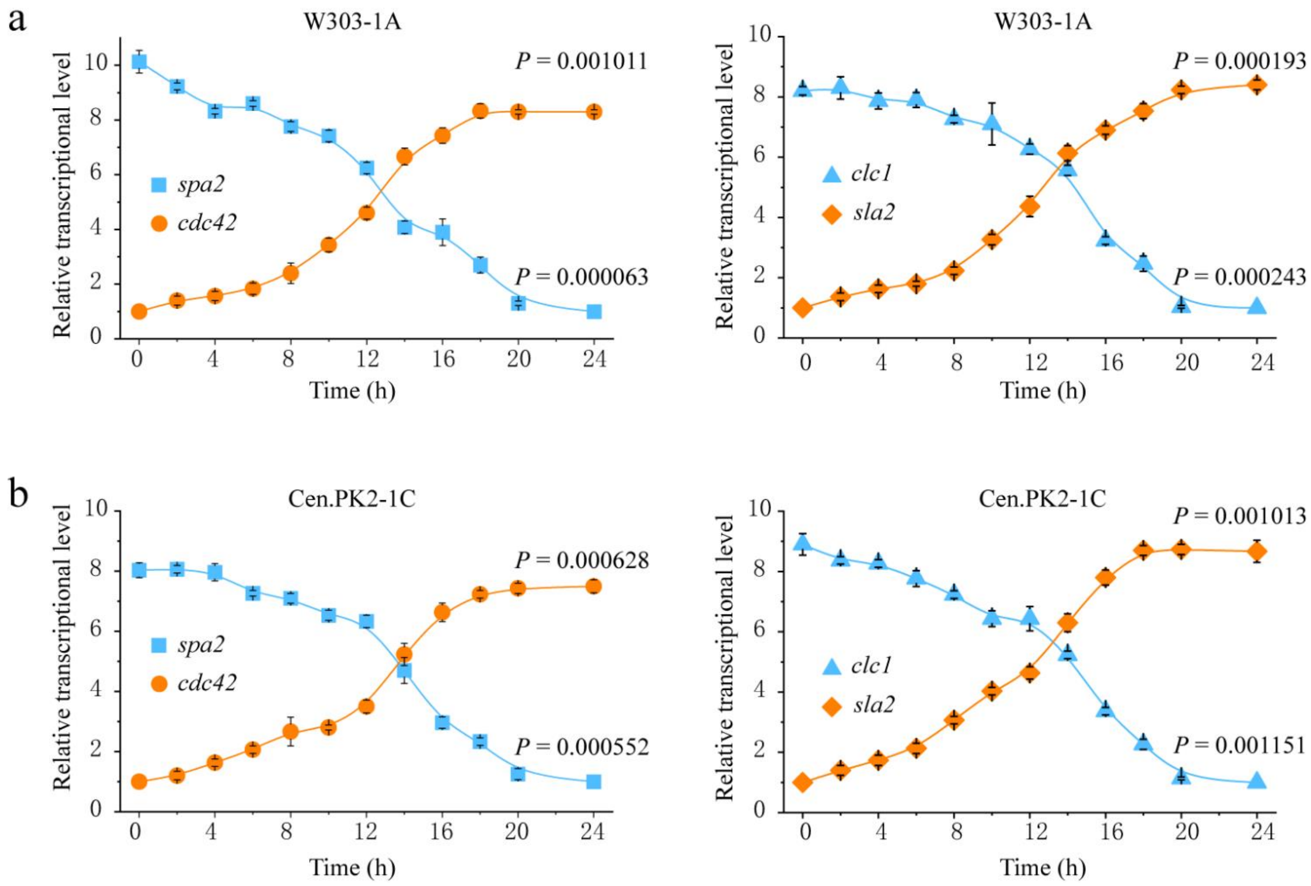


**Supplementary Figure 20. The universal properties of ABSC that regulate gene expression in a two-phase manner were confirmed in strain W303-1A (a) and Cen.PK2-1C (b).** *P* values are from a Student's two-sided t-test of the difference between 24 h and 0 h. Values in Fig.S20 are shown as mean ± S.D. from three (n = 3) biological replicates. Source data are provided as a Source Data file.

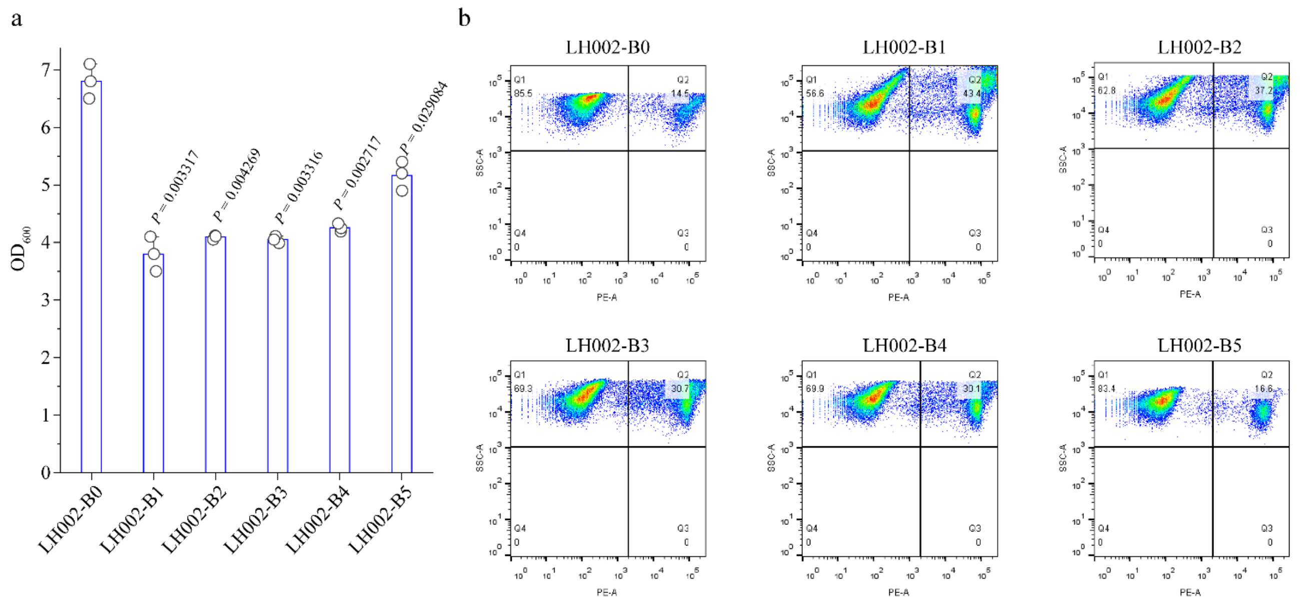


**Supplementary Figure 21**. **The cell growth of LH002-B0 to LH002-B5. a**, the cell density ($OD_{600}$) of each strain; Values are shown as mean ± S.D. from three (n = 3) biological replicates. **b**, the population distribution of cell viability in strain LH002-B0, LH002-B1, LH002-B2, LH002-B3, LH002-B4, and LH002-B5, which were analyzed by flow cytometry. *P* values are from a Student's two-sided t-test of the difference of control strain. Source data are provided as a Source Data file.

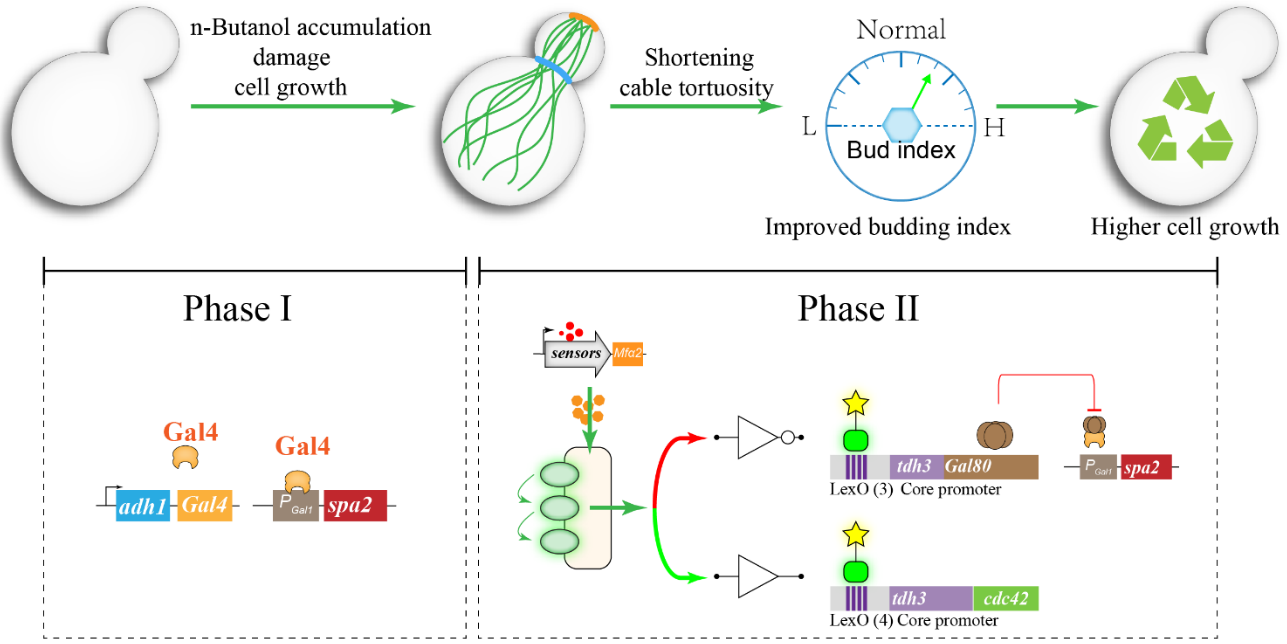


**Supplementary Figure 22. Key problems and the corresponding solution in the process of n-butanol production.**

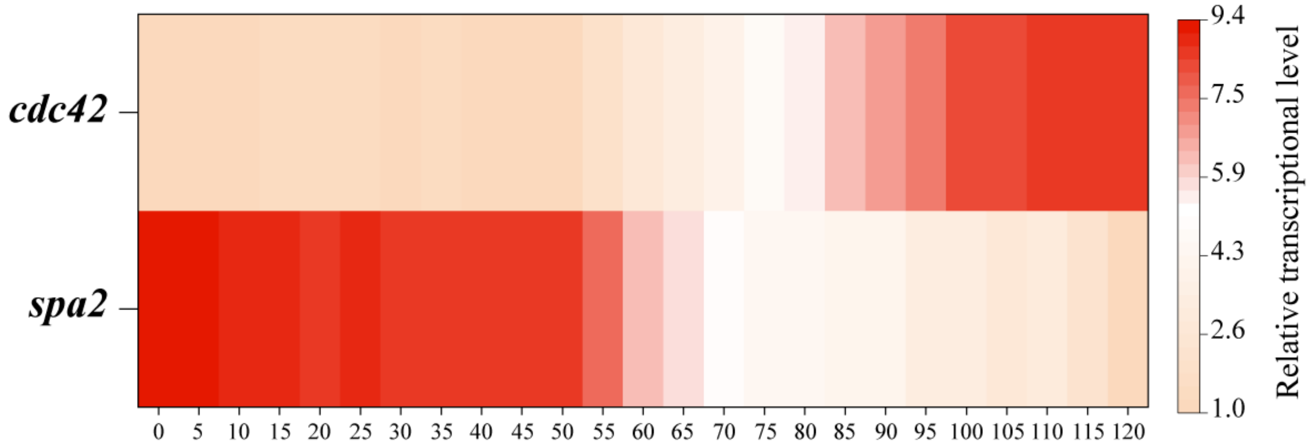


**Supplementary Figure 23. The gene expression level in strain LH002-B5 was confirmed by RT-PCR.** In strain LH002-B5, the expression of *SPA2* was activated during the first 50 h, whereas the expression of *spa2* was started to be repressed and *CDC42* was activated from 50-120 h of the butanol fermentation process by strain LH002-B5. Source data are provided as a Source Data file.

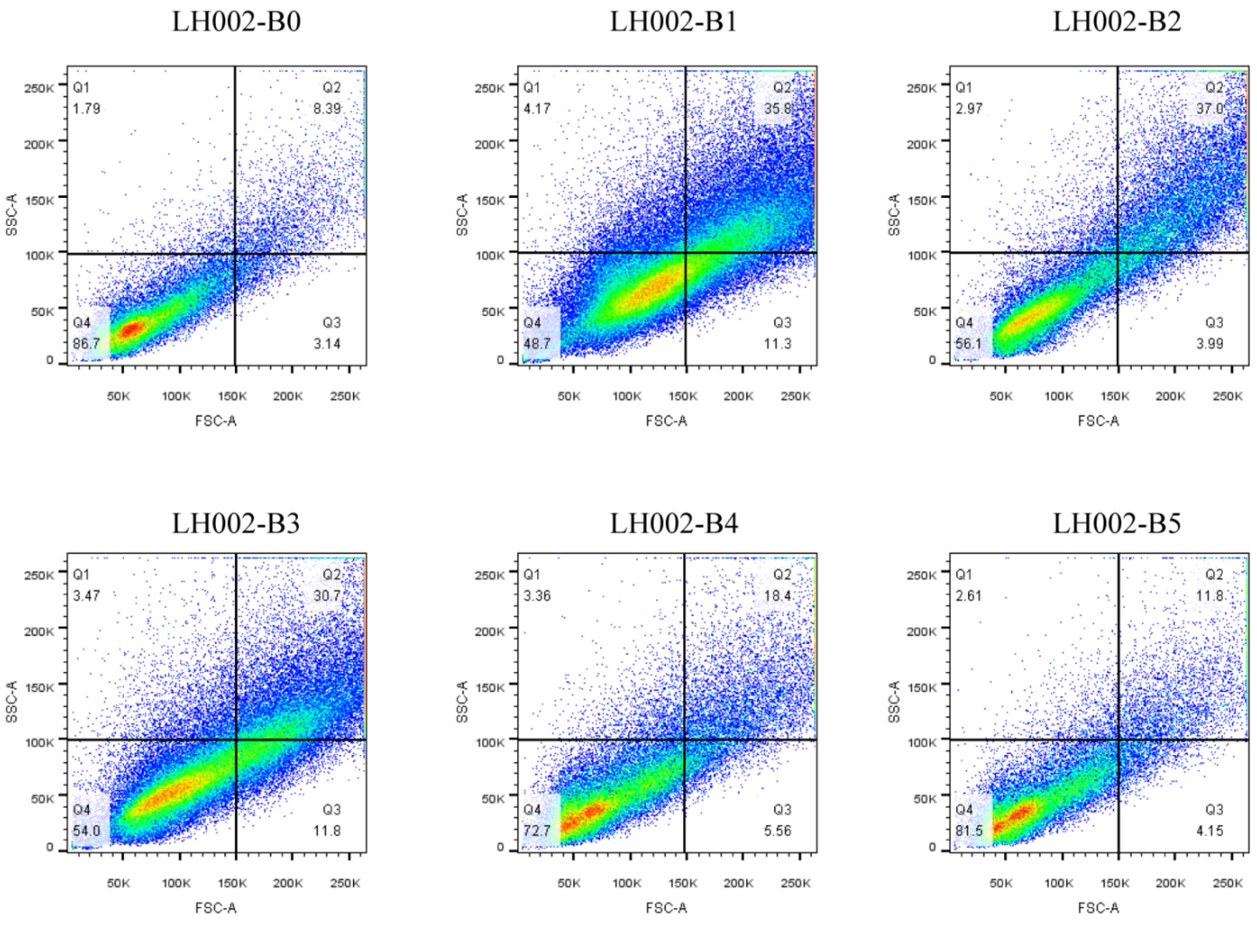


**Supplementary Figure 24**. **The population distribution of normal yeast morphology in LH002-B0, LH002-B1, LH002-B2, LH002-B3, LH002-B4, and LH002-B5.** The morphology was characterized by SSC v.s. FSC density plot by cell cytometry and 20,000 cells were collected. Each dot or point on the plot represents an individual cell that has passed through the laser. Source data are provided as a Source Data file.

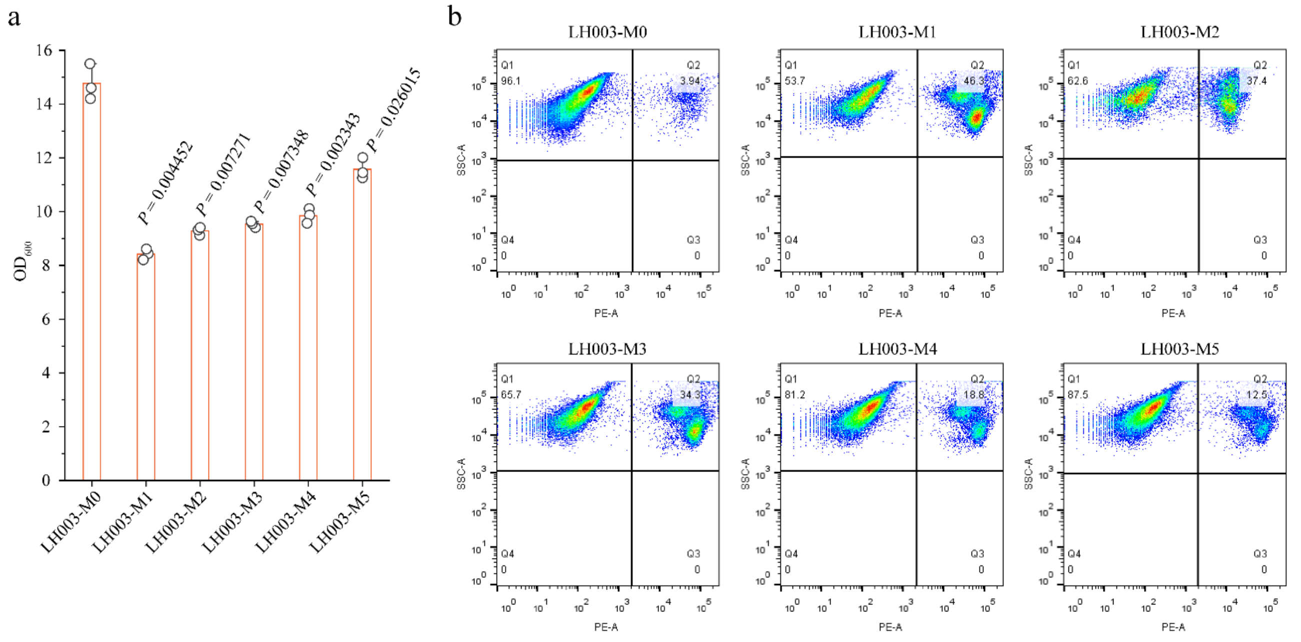


**Supplementary Figure 25**. **The cell growth of LH003-M0 to LH003-M5. a**, the cell density ($OD_{600}$) of each strain; Values are shown as mean ± S.D. from three (n = 3) biological replicates. **b**, the population distribution of cell viability in strain LH003-M0, LH003-M1, LH003-M2, LH003-M3, LH003-M4, and LH003-M5, which were analyzed by flow cytometry. *P* values are from a Student's two-sided t-test of the difference of control strain. Source data are provided as a Source Data file.

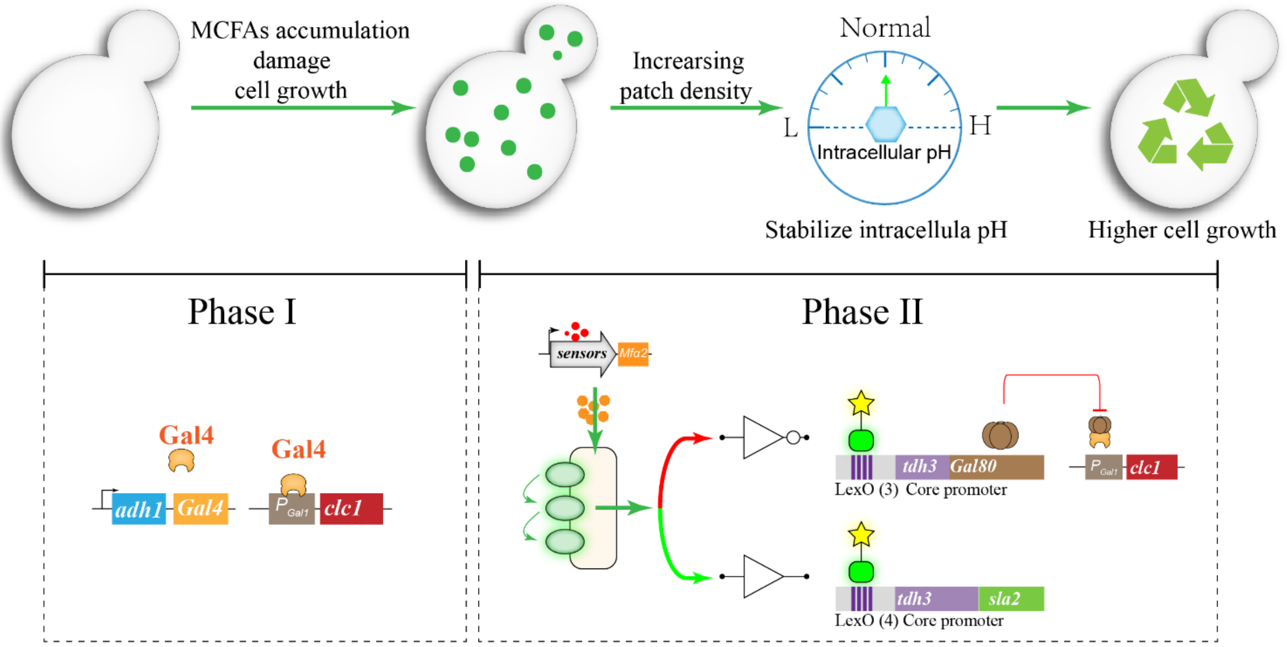


**Supplementary Figure 26. Key problems and the corresponding solution in the process of MCFAs production.**

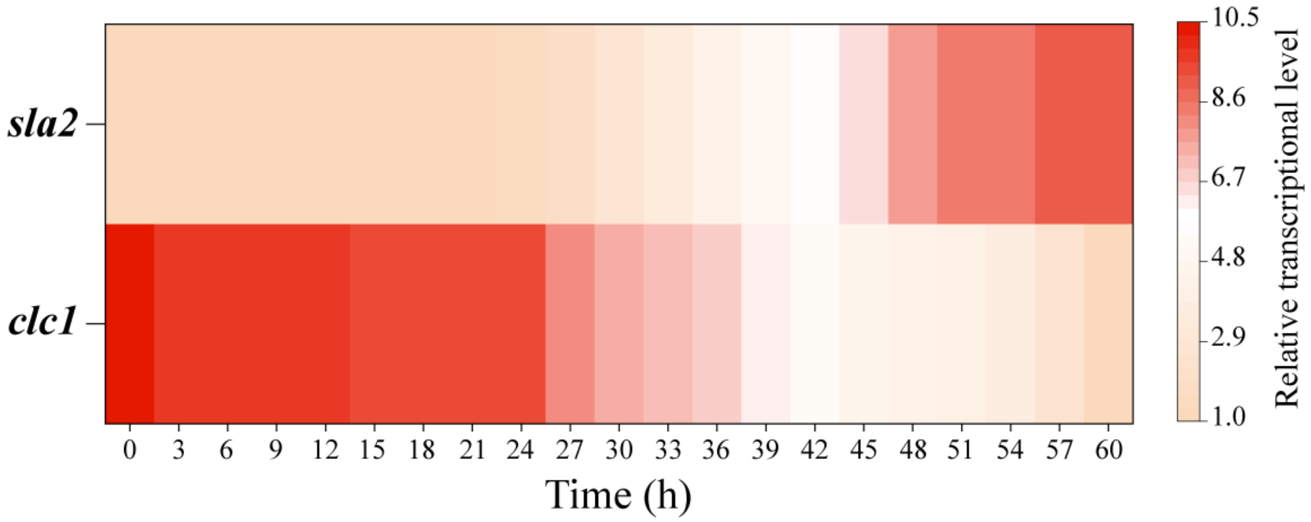


**Supplementary Figure 27. The gene expression level in strain LH003-M5 was confirmed by RT-PCR.** In strain LH003-M5, the expression of *CLC1* was activated under the Gal1 promoter at the first 40 h, whereas the expression of *SLA2* was activated from 40 to 100 h. Source data are provided as a Source Data file.

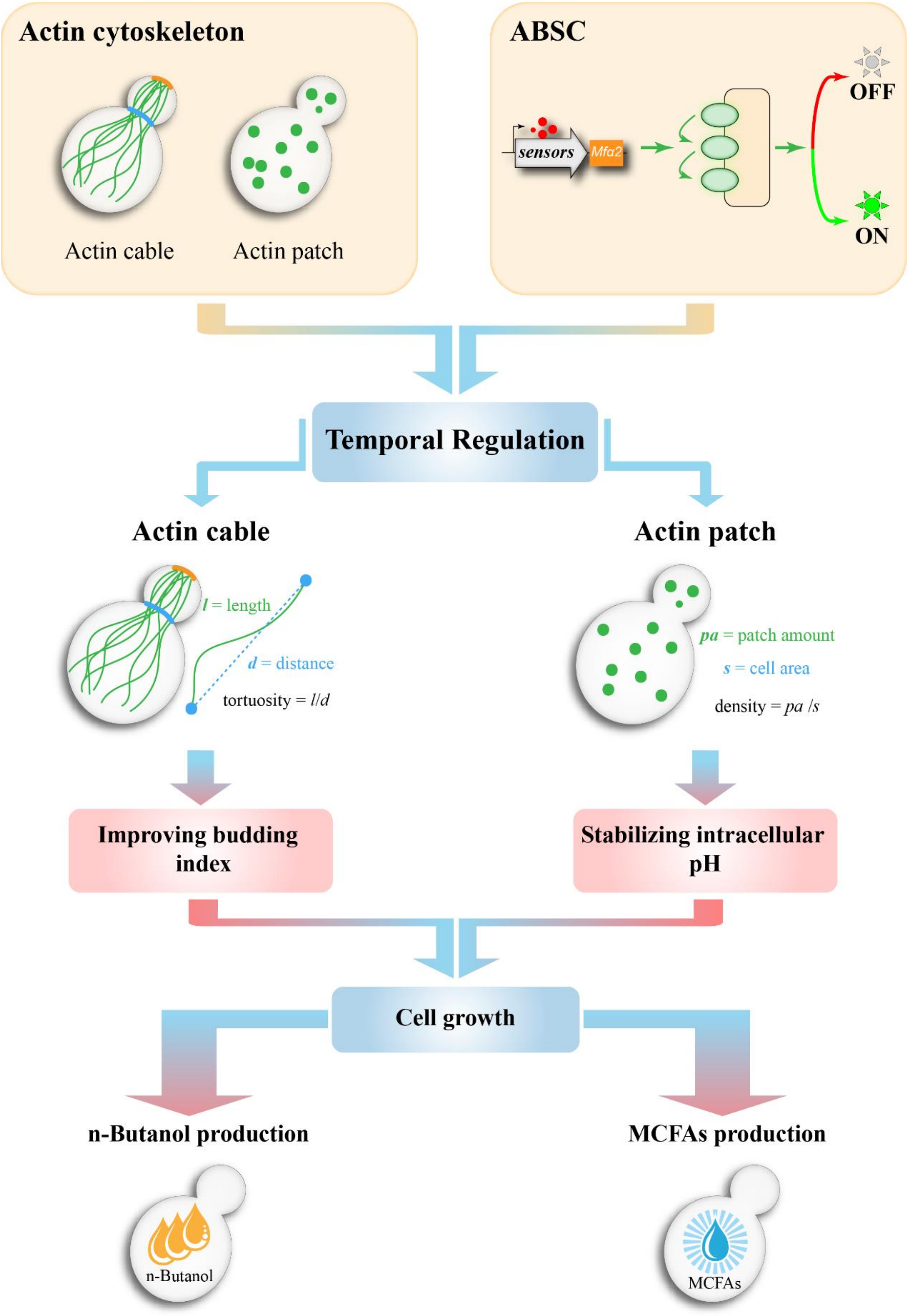


**Supplementary Figure 28. The framework of this study.**

**Supplementary Table 1. Strategies and effect of some classical examples to alleviate the toxicity of biofuels.**

| No. | Strategies | Effect | Ref. |
|---|---|---|---|
| 1 | Deletion of *GLN3*, a transcriptional activator in nitrogen catabolite repression system. | Deleting *GLN3* could enhance tolerance and boost isobutanol production 4.9-fold in *S. cerevisiae*. | 1 |
| 2 | Overexpression of *ESBP6*, a protein that similar to monocarboxylate permeases | Increased the tolerance to aromatic amino acid and coumaric acid. | 2 |
| 3 | Overexpression of *TPO1* and its mutant, an efflux pump | Improved the tolerance to medium-chain fatty acids and its production increased by 3.2-fold. | 3 |
| 4 | Overexpression of *SECB*, a chaperone. | Maximum butanol tolerance of *E. coli* was increased to 1.8% (v/v) n-butanol. | 4 |
| 5 | Deletion of *ATG22*, a gene that belongs to the autophagy-related genes family | Increased stress tolerance to acetic acid. | 5 |

## Supplementary references


1. Kuroda, K. et al. Critical roles of the pentose phosphate pathway and GLN3 in isobutanol-specific tolerance in yeast. *Cell Syst* **9**, 534-547 (2019).
2. Pereira, R. et al. Elucidating aromatic acid tolerance at low pH in *Saccharomyces cerevisiae* using adaptive laboratory evolution. *Proc Natl Acad Sci U S A* **117**, 27954-27961 (2020).
3. Zhu, Z. et al. Multidimensional engineering of *Saccharomyces cerevisiae* for efficient synthesis of medium-chain fatty acids. *Nat Catal* **3**, 64-74 (2020).
4. Xu, G., Wu, A., Xiao, L., Han, R. & Ni, Y. Enhancing butanol tolerance of *Escherichia coli* reveals hydrophobic interaction of multi-tasking chaperone SecB. *Biotechnol Biofuels* **12**, 164-176 (2019).
5. Hu, J. et al. Deletion of Atg22 gene contributes to reduce programmed cell death induced by acetic acid stress in *Saccharomyces cerevisiae*. *Biotechnol Biofuels* **12**, 298-317 (2019).
