# Supplementary material for: Enhancing biofuels production by engineering the actin cytoskeleton in Saccharomyces cerevisiae
Source: Nat Commun. 2022 Apr 7;13:1886. doi: 10.1038/s41467-022-29560-6 (PMC8991263; doi:10.1038/s41467-022-29560-6)
Supplement: Supplementary file 2 — Description of Additional Supplementary Files [file 41467_2022_29560_MOESM2_ESM.pdf]

Description of Additional Supplementary Files

File name: Supplementary Data 1

Description: Strains used in this study.

File name: Supplementary Data 2

Description: Plasmids used in this study.

File name: Supplementary Data 3

Description: Codon-optimized genes used in this study.

File name: Supplementary Data 4

Description: Primers used in this study.
