# Supplementary material for: Enhancing biofuels production by engineering the actin cytoskeleton in Saccharomyces cerevisiae
Source: Nat Commun. 2022 Apr 7;13:1886. doi: 10.1038/s41467-022-29560-6 (PMC8991263; doi:10.1038/s41467-022-29560-6)
Supplement: Supplementary file 7 — Reporting Summary [file 41467_2022_29560_MOESM7_ESM.pdf]

nature portfolio

Corresponding author(s): Liming Liu

Last updated by author(s): Mar 12, 2022

Reporting Summary

Nature Portfolio wishes to improve the reproducibility of the work that we publish. This form provides structure for consistency and transparency in reporting. For further information on Nature Portfolio policies, see our Editorial Policies and the Editorial Policy Checklist.

Statistics

For all statistical analyses, confirm that the following items are present in the figure legend, table legend, main text, or Methods section.

n/a	Confirmed	
[ ]	[x]	The exact sample size (*n*) for each experimental group/condition, given as a discrete number and unit of measurement
[ ]	[x]	A statement on whether measurements were taken from distinct samples or whether the same sample was measured repeatedly
[ ]	[x]	The statistical test(s) used AND whether they are one- or two-sided<br>*Only common tests should be described solely by name; describe more complex techniques in the Methods section.*
[ ]	[x]	A description of all covariates tested
[x]	[ ]	A description of any assumptions or corrections, such as tests of normality and adjustment for multiple comparisons
[ ]	[x]	A full description of the statistical parameters including central tendency (e.g. means) or other basic estimates (e.g. regression coefficient) AND variation (e.g. standard deviation) or associated estimates of uncertainty (e.g. confidence intervals)
[ ]	[x]	For null hypothesis testing, the test statistic (e.g. *F*, *t*, *r*) with confidence intervals, effect sizes, degrees of freedom and *P* value noted<br>*Give P values as exact values whenever suitable.*
[x]	[ ]	For Bayesian analysis, information on the choice of priors and Markov chain Monte Carlo settings
[x]	[ ]	For hierarchical and complex designs, identification of the appropriate level for tests and full reporting of outcomes
[x]	[ ]	Estimates of effect sizes (e.g. Cohen's *d*, Pearson's *r*), indicating how they were calculated

Our web collection on statistics for biologists contains articles on many of the points above.

Software and code

Policy information about availability of computer code

Data collection	SpectraMax M3 plate reader, ChemiScope 6000, IBright FL1000, BD FACSArica III cytometer , NanoDrop ND-1000 UV-Vis Spectrophotometer, gas chromatography (GC-2014SBA- 90E biological sensor, Nikon eclipse 80i microscope, FEI Company Quanta-200 and H-7650.
Data analysis	Origin 2019 64 bit, Microsoft Excel 2016, SoftMax pro 6.5, FlowJo_V10, SPSS R24.0.0.0, Adobe Illustrate CS6, Snapgene 1.1.3, Primer 7.0 Microsoft Word 2016, Nikon eclipse 80i microscope, DNAMAN 6.0, Image J 1.63.

For manuscripts utilizing custom algorithms or software that are central to the research but not yet described in published literature, software must be made available to editors and reviewers. We strongly encourage code deposition in a community repository (e.g. GitHub). See the Nature Portfolio guidelines for submitting code & software for further information.

Data

Policy information about availability of data

All manuscripts must include a data availability statement. This statement should provide the following information, where applicable:

- Accession codes, unique identifiers, or web links for publicly available datasets
- A description of any restrictions on data availability
- For clinical datasets or third party data, please ensure that the statement adheres to our policy

Data supporting the findings of this work are available within the paper and its Supplementary Information files. A reporting summary for this Article is available as a Supplementary Information file. Source data are provided with this paper, which is also available at Figshare [https://doi.org/10.6084/m9.figshare.19329092.v1]. Source data are provided with this paper.

Field-specific reporting

Please select the one below that is the best fit for your research. If you are not sure, read the appropriate sections before making your selection.

- [x] Life sciences
- [ ] Behavioural & social sciences
- [ ] Ecological, evolutionary & environmental sciences

For a reference copy of the document with all sections, see nature.com/documents/nr-reporting-summary-flat.pdf

Life sciences study design

All studies must disclose on these points even when the disclosure is negative.

Sample size	No sample-size calculations were performed. As indicated in the text, all experiments were performed from a single colony, which provides some limited information about the distribution of the measurements and is typical of similar experiments and studies in the field. For flow cytometry, we analyzed 10000 cells / sample.
Data exclusions	No data were excluded from the analyses.
Replication	All the biochemical and biological experiments were performed in three replicated or more. Data were repeatable on different date.
Randomization	Pipet tips were used to scoop few cells for culturing, the scooping locations are all random.
Blinding	Blinding is not relevant to our study because none of our data is based on qualitative scoring metrics nor does it involve animals or human research participants. As described in the above section for randomization, blinding during group allocation is irrelevant because the samples of bacterial cultures that were split into different conditions were random samplings and there is no control over which cells will be selected and thus, no bias during group allocation.

Reporting for specific materials, systems and methods

We require information from authors about some types of materials, experimental systems and methods used in many studies. Here, indicate whether each material, system or method listed is relevant to your study. If you are not sure if a list item applies to your research, read the appropriate section before selecting a response.

Materials & experimental systems

n/a	Involved in the study
x	Antibodies
x	Eukaryotic cell lines
x	Palaeontology and archaeology
x	Animals and other organisms
x	Human research participants
x	Clinical data
x	Dual use research of concern

Methods

n/a	Involved in the study
x	ChIP-seq
	x Flow cytometry
x	MRI-based neuroimaging

Flow Cytometry

Plots

Confirm that:

- [x] The axis labels state the marker and fluorochrome used (e.g. CD4-FITC).
- [x] The axis scales are clearly visible. Include numbers along axes only for bottom left plot of group (a 'group' is an analysis of identical markers).
- [ ] All plots are contour plots with outliers or pseudocolor plots.
- [x] A numerical value for number of cells or percentage (with statistics) is provided.

Methodology

Sample preparation	For a fluorescence assay, we diluted cells 1:150 into PBS (phosphate-buffered solution) and ran them on a BD FACS AriaTM III apparatus cell analyzer (BD Biosciences).
Instrument	BD FACS AriaTM III apparatus cell analyzer (BD Biosciences).
Software	FlowJo (version 10) and FACS Diva for analysis

Cell population abundance	20,000 cells for each sample
Gating strategy	The assays were performed using a LSR Fortessa instrument (BD Biosciences) using FITC and PE-TxRed channels. The voltage gains for each detector were set to FITC, 500V and PE-TxRed, 650 V. For each sample, at least 20,000 counts were recorded using a 0.5 mL•s−1 flow rate.All data were exported in FCS3 format and processed using FlowJo software (FlowJo, LLC). A gate was previously designed based on forward and side scatter.

☒ Tick this box to confirm that a figure exemplifying the gating strategy is provided in the Supplementary Information.
